# Vinburnine potentiates anti-PD1 immunotherapy in melanoma through IL-24 secretion via P38/MAPK/ATF3 signaling

**DOI:** 10.1186/s13046-025-03521-5

**Published:** 2025-08-27

**Authors:** Susi Zhu, Xu Zhang, Waner Liu, Zhe Zhou, Siyu Xiong, Xiang Chen, Cong Peng

**Affiliations:** 1 Department of Dermatology, Xiangya Hospital, Central South University, Changsha, Hunan China; 2 National Engineering Research Center of Personalized Diagnostic and Therapeutic Technology, Xiangya Hospital, Central South University, Changsha, Hunan China; 3 Furong Laboratory, Central South University, Changsha, Hunan China; 4 Hunan Key Laboratory of Skin Cancer and Psoriasis, Human Engineering Research Center of Skin Health and Disease, Xiangya Hospital, Central South University, Changsha, Hunan China; 5 National Clinical Research Center for Geriatric Disorders, Xiangya Hospital, Central South University, Changsha, Hunan China

**Keywords:** Melanoma, Vinburnine, Immunotherapy, IL-24, CD8^+^ t cells

## Abstract

**Background:**

Melanoma, a highly aggressive and immunogenic skin cancer, often develops resistance to immunotherapy due to the immunosuppressive tumor microenvironment (TME). Although PD-1/PD-L1 inhibitors have significantly improved treatment outcomes, 30%-40% of patients exhibit no response or develop resistance. Mechanisms such as T-cell exhaustion within the TME limit therapeutic efficacy, necessitating the exploration of novel strategies to enhance immune responses.

**Methods:**

This study evaluated the effects of Vinburnine (Vin) on melanoma cell proliferation, migration, invasion, apoptosis, and DNA damage through in vitro experiments. Transcriptomic analysis, Western blot, RT-PCR, dual-luciferase reporter assays, and ChIP experiments revealed the mechanism by which Vin regulates IL-24 via ATF3. The anti-tumor efficacy of Vin or IL-24 in combination with PD-1 monoclonal antibody, as well as their modulation of the tumor microenvironment, were validated through luciferase-mediated cytotoxicity assays and a murine melanoma model. Additionally, the correlation between IL-24 expression and patient prognosis or immunotherapy response was analyzed using public databases.

**Results:**

This study delineates the phenotypic mechanisms by which vinburnine suppresses melanoma proliferation. Vin induces reactive oxygen species (ROS) generation, leading to DNA damage and the subsequent activation of the apoptotic cascade in melanoma cells. Additionally, vinburnine activates the P38/MAPK/ATF3 signaling axis, which drives the secretion of interleukin-24 (IL-24), enhancing the functionality of CD8^+^ T cells and modulating the tumor immune microenvironment to favor antitumor immunity. Notably, the combination of vinburnine with anti-PD-1 antibody therapy produces synergistic effects, effectively addressing certain limitations of current immunotherapeutic approaches.

**Conclusions:**

These findings underscore the therapeutic potential of vinburnine, particularly when used in combination with immune checkpoint inhibitors, as a promising strategy for melanoma treatment.

**Supplementary Information:**

The online version contains supplementary material available at 10.1186/s13046-025-03521-5.

## Background

In recent years, cancer immunotherapy has emerged as a pivotal strategy for cancer treatment, demonstrating remarkable efficacy, particularly in melanoma. Immune checkpoint inhibitors (ICIs), especially anti-PD-1/PD-L1 antibodies, block tumor immune evasion mechanisms and re-activate the immune system to recognize and eliminate tumor cells, achieving groundbreaking success in melanoma therapy [1–3]. However, the effectiveness of immunotherapy remains limited by several challenges. Approximately 30–40% of patients show no response to monotherapy with PD-1 inhibitors, and some patients who initially respond well may develop acquired resistance over time. Consequently, the overall objective response rate remains suboptimal [4–6]. The TME exhibits immunosuppressive features, such as T cell exhaustion, the accumulation of immune-suppressive cells, elevated levels of inflammatory mediators, and altered metabolic processes, all of which hinder the effectiveness of ICIs in antitumor responses [7]. Furthermore, the complex immune evasion mechanisms of melanoma exacerbate the limitations of current therapies [8]. Therefore, effectively activating the immune system and improving the therapeutic response to immune checkpoint inhibitors are critical research priorities and pressing challenges in the field.

CD8^+^ T cells are the key effector cells in the tumor immune response, capable of directly killing tumor cells by recognizing tumor antigens [9]. In the TME of melanoma, CD8^+^ T cells are frequently in a state of functional exhaustion or anergy. Their cytotoxic activity is dampened by immune checkpoint signaling, particularly through the PD-1/PD-L1 axis, and by inhibitory factors secreted by tumor cells, such as TGF-β and IL-10 [10, 11]. Recent studies have highlighted the importance of modulating the interactions between tumor cells and immune cells within the TME to enhance the response to immunotherapy [12, 13]. For example, inducing tumor cells to secrete immune-regulatory cytokines, such as IL-15, can promote the proliferation and functional activation of CD8^+^ T cells, significantly enhancing their antitumor efficacy [14, 15]. This strategy provides a new research direction and therapeutic approach to improve the effectiveness of melanoma immunotherapy. Therefore, enhancing CD8^+^ T cell activity is essential for counteracting the immunosuppressive effects within the TME and boosting the efficacy of ICIs.

Recently, natural products have emerged as promising candidates to serve as adjuvants, potentially improving the therapeutic outcomes of ICIs. Studies have shown that certain natural compounds can modulate the TME or boost immune responses, significantly improving the therapeutic effects of ICIs [16, 17]. Plant extracts and natural compounds, such as resveratrol and curcumin, have demonstrated sensitizing effects in tumor immunotherapy and show promising clinical potential. These natural products enhance the efficacy of tumor immunotherapy by promoting T cell function, inhibiting immunosuppressive cell activity, and modulating other immune-related mechanisms [18, 19]. Several clinically approved natural products have shown promise in tumor immunotherapy. For instance, anticancer drugs like paclitaxel, which primarily exert antitumor effects through direct cytotoxic actions on tumor cells, have also attracted increasing attention for their immune-modulatory properties [20]. Recent studies have demonstrated that the hypolipidemic drug bezafibrate, when combined with CTLA-4 monoclonal antibody therapy, synergistically inhibits melanoma growth [21]. Furthermore, the administration of recombinant murine IL-17 A in combination with dual checkpoint blockade targeting CTLA-4 and PD-1 significantly enhances CD8^+^ T cell infiltration and effectively suppresses melanoma progression [22]. In this context, our research focuses on vinburnine, an alkaloid derived from the *Catharanthus genus*, primarily used in the treatment of neurological and cardiovascular diseases. Although researchers have extensively studied the role of vinburnine in these areas, its potential and mechanisms in cancer remain unclear. Preliminary research suggests that vinburnine may have therapeutic effects on head and neck squamous cell carcinoma and hepatocellular carcinoma [23, 24]. However, it remains unclear whether vinburnine can suppress the malignant phenotype of melanoma or enhance antitumor immune response. Therefore, further investigation into the regulatory effects of vinburnine on tumor immunity, particularly in combination with immune checkpoint inhibitors, may provide new therapeutic strategies for melanoma and other cancers in immunotherapy.

## Methods

### Reagents, plasmids, cell transfection, and viral infection procedures

Vinburnine (Vin), N-acetylcysteine (NAC), and SB203580 were purchased from MedChemExpress (MCE, Wuhan, China). The cell stimulation cocktail was obtained from eBioscience (San Diego, CA, USA). The plasmids (shATF3, shIL-24 and shIL-20R2) were purchased from GeneChem (Shanghai, China). The pCCLc-MNDU3-Luciferase-PGK-EGFP-WPRE vector was obtained from Addgene (Watertown, MA, USA), while the ATF3 overexpression plasmid was sourced from Vigene Biosciences Co., Ltd (Jinan, Shandong, China). For lentiviral vector production, HEK-293T cells were plated in 10 cm culture dishes and transfected with TurboFect transfection reagent (Thermo Fisher Scientific, Waltham, MA, USA), following the manufacturer’s instructions. The plasmid mixture, consisting of the target lentiviral plasmid, pspAX2, and pMD2G, was prepared at a mass ratio of 4:3:1. After 24 h of incubation, the medium was replaced with fresh complete culture medium. Lentiviral-containing supernatants were collected at 48 h and 72 h post-transfection, pooled and filtered through a 0.45 μm filter to eliminate cell debris. For viral infection, melanoma cells were incubated with the filtered viral supernatants, supplemented with polybrene (10 µg/mL) for 24 h. Infected cells were then selected using puromycin and used for subsequent experiments.

### Cell culture

A375, SK-MEL-28, SK-MEL-5, and HEK-293T cells were obtained from American Type Culture Collection (ATCC, Manassas, VA, USA) and maintained in DMEM (Gibco, Pittsburgh, PA, USA), 10% Fetal Bovine Serum (FBS) (ExCell Bio, Shanghai, China), and 1% P/S (BI, Beit Haemek, Israel). B16F10 and Jurkat cells were sourced from the ATCC and cultured in RPMI 1640 (Gibco), supplemented with 10% FBS and P/S as described above. All cells were cultured in a humidified, 37 °C incubator with a gaseous environment of 5% CO2.

### Cell viability and colony formation assay

3 × 10^3^ cells/well were plated in 96-well plates and cultured overnight; Vin was diluted to serial concentration in the medium and added to the well. Cell viability assay was assessed with Cell Counting Kit-8 (CCK-8) (Selleck, Shanghai, China) by a microplate at 450 nm reader after 24, 48, and 72 h of exposure to a serial dilution of Vin (2.5–20 µM), with DMSO used as the vehicle control. To assess cell colony formation, a total of 1 × 10^3^ cells/well were plated in 6-well plates and cultured overnight, and were incubated with Vin-free DMEM medium along with various concentrations of Vin for 48 h. Subsequently, the medium was replaced, and the cells were incubated for 14 days. The colonies were then fixed in 4% formaldehyde and stained with 0.1% crystal violet. After staining, the colonies were counted and analyzed using ImageJ software.

### Cell migration and invasion assays

Cell migration and invasion were measured using wound healing and Transwell assays. In the wound healing assay, 8 × 10^5^ cells were seeded into 6-well plates and allowed to adhere overnight. A linear scratch wound was then created using a micropipette tip, and the cells were treated with Vin for 48 h. Images of cell migration were captured at 0, 24, and 48 h. For the Transwell assay, 5 × 10^4^ cells in 100 µL of serum-free DMEM were placed in the upper chamber (8 μm, Corning, NY, USA) with or without Matrigel (BD Biosciences, Franklin Lakes, NJ, USA). The bottom chamber was filled with 500 µL of DMEM containing 30% FBS as a chemoattractant. After 12 and 24 h of Vin treatment, cells that had migrated or invaded were collected, fixed, and stained with crystal violet. The cells were then photographed using an inverted microscope, and the number of migrated cells was quantified using ImageJ software.

### Reactive oxygen species (ROS) detection

Cellular ROS levels were measured by using the DCFH-DA ROS assay kit (Solarbio, Beijing, China) according to the manufacturer’s protocol. In short, A375, SK-MEL-28, and SK-MEL-5 cells treated with Vin were washed with buffer solution when the appropriate density was reached. Then, the cells were stained with 10 µM DCFH-DA at 37 ℃ for 30 min without light. After washing with buffer solution, the samples were detected by flow cytometry.

### Comet assay

The comet assay was conducted according to the protocol provided by the manufacturer (Beyotime, Shanghai, China). Briefly, A375, SK-MEL-28, and SK-MEL-5 cells were treated with or without Vin for 24 h, followed by cell collection and washing with phosphate-buffered saline (PBS). A total of 10^4^ cells in 10 µL were mixed with 75 µL of agarose. Transfer the mixture onto a comet slide (Beyotime) and let it solidify at 4 ℃ for 10 min. Cells were lysed at 4°C for 1 h and then subjected to dark electrophoresis using cold alkaline electrophoresis buffer for 30 min. The comet slide was incubated in a neutral buffer at 4°C for 10 min, followed by the removal of the buffer. The slide was stained with propidium iodide solution in the dark for 15 min and examined under a fluorescence microscope. Quantification was performed using CASP software.

### Cell cycle and apoptosis analysis

To assess the impact of Vin treatment on melanoma cell cycle progression and apoptosis, A375, SK-MEL-28, and SK-MEL-5 cell lines were cultured in a complete medium and exposed to Vin at concentrations of 2.5 and 5 µM for 24 h. Following treatment, cells were harvested by trypsinization, washed with PBS, and fixed in 70% ethanol at -20 °C for at least 2 h. For cell cycle analysis, the fixed cells were stained with propidium iodide (PI) at 50 µg/mL in the presence of RNase A (100 µg/mL) to digest RNA. The cells were then incubated at 37 °C for 30 min in the dark. Cell cycle distribution was analyzed by flow cytometry using a BD FACSymphony, and the data were processed with FlowJo software. For apoptosis detection, treated cells were resuspended in a binding buffer and stained with Annexin V-FITC and PI, following the manufacturer’s protocol (Beyotime). Flow cytometric analysis was performed on a BD FACSymphony, and apoptotic cell percentages were determined based on Annexin V^+^PI^−^ (early apoptosis) and Annexin V^+^PI^+^ (late apoptosis). All experiments were performed in triplicate, and results are presented as the mean ± standard deviation (SD).

### RNA isolation and quantitative real-time PCR (qRT-PCR)

Total RNA from melanoma cells and Jurkat cells samples were extracted using MagZol reagent (Guangzhou, Guangdong, China) according to the manufacturer’s protocol, and cDNAs were synthesized using the Hifair^®^ Ⅲ kit (Yeasen, Shanghai, China). Then, Quantitative real-time PCR assays were conducted using SYBR Green qPCR Master Mix (Selleck) with QuantStudio 3 Real-Time PCR System (Thermo Fisher Scientific) to assess the gene expression. The gene expression was normalized to β-actin mRNA expression using the ΔCt method (2^–ΔΔCt^) and expressed as a ratio relative to the untreated control. All primers used are listed in table S1a and table S1b.

### Western blotting

As previously described [25]. The primary antibodies were diluted as follows: anti-p-ATM (1:1000, CST, #5883s, Boston, USA), anti-p-ATR (1:1000, CST, #2853s), anti-γH2AX (1:1000, CST, #9718s), anti-ATF3 (1:1000, Abcam, ab207434), anti-Bcl2 (1:1000, Proteintech, #12789, Chicago, USA), anti-BAX (1:1000, Proteintech, #50599), anti-Cleaved Caspase-3 (1:1000, CST, #9664S), anti-caspase-9 (1:1000, Proteintech, #66169), anti-p-JNK (1:1000, Proteintech, #80024), anti-JNK (1:200, Santa Cruz, sc-7345, Biotechnology, CA, USA), anti-p-ERK1/2 (1:1000, Proteintech, #28733), anti-ERK (1:1000, Proteintech, #11257), anti-p-P38 (1:1000, Proteintech, #28796), anti-P38 (1:200, Santa Cruz, sc-7972), anti-IL-24 (1:1000, Proteintech, #26772), anti-β-Actin (1:5000, Proteintech, #66009). The bands were visualized with a gel documentation system (Bio-Rad, Hercules, California, USA), and row values were quantified by ImageJ.

### Transcriptome sequencing (RNA-seq)

RNA-seq was performed on cells treated with vinburnine (2.5 µM and 5 µM) and untreated controls, with three biological replicates per group. The analysis was conducted using Illumina technology by Wuhan Huada Gene Company. Genes with a fold change greater than 2 and a *P*-value below 0.05 were identified as differentially expressed.

### Enzyme-linked immunosorbent assay (ELISA)

The levels of interleukin-24 (IL-24) in the cell culture supernatant (Solarbio) and tumor tissues from mice (Solarbio) were determined using a commercially available ELISA kit, following the manufacturer’s instructions.

### Dual luciferase reporter assay

The IL-24 core promoter region was identified and cloned into the pGL3 Basic vector (Promega Corporation, Madison, WI, USA). TurboFect Transfection Reagent was used to transfect the plasmids according to the manufacturer’s instructions. At the same time, the cells were treated with or without Vin. The cells were collected 48 h post-transfection to assess luciferase activity. The measurement was performed using a dual luciferase reporter assay system (Promega Corporation) according to the manufacturer’s protocol, with results normalized to renilla luciferase activity.

### Chromatin immunoprecipitation (ChIP)

SimpleChIP^®^ Enzymatic Chromatin IP Kit (CST) was used to verify the interaction between ATF3 protein and IL-24 promoter. Briefly, SK-MEL-28 cells were treated with Vin (2.5 and 5µM) for 48 h. DNA and proteins were cross-linked by incubation with 1% formaldehyde at room temperature for 10 min, lysed and sonicated to obtain chromatin fragments. Then chromatin fragments with about 150–900 bp base pairs of DNA in size were prepared by sonication, incubated with IgG and anti-ATF3 (1:100, Abcam, ab207434) antibody overnight at 4 °C, and precipitated with protein G magnetic beads. After cross-linking reversal, qRT-PCR was used to detect the DNA template enrichment with the primers in Table S2.

### In vivo experiments

All experimental procedures were performed in strict adherence to the National Research Council’s Guide for the Care and Use of Laboratory Animals. All animal studies were conducted with approval from the Ethics Committee at Xiangya Hospital, adhering to established ethical standards. Mouse melanoma B16F10 cells (5 × 10^5^ cells/mouse) were subcutaneously injected into 8-week-old C57BL/6 female mice (Hunan SJA Laboratory Anima, Changsha, Hunan, China). Once the tumor volume reached approximately 50–100 mm^3^, the mice were randomly assigned into the vehicle group (0.5% carboxymethylcellulose (CMC), Aladdin, Shanghai, China), Vin (10 mg/kg) and Vin (20 mg/kg) treatment groups. Vin was orally administrated once daily for 10 days.

For combination treatment, mice were treated with one of the following regimens: isotype antibodies, anti–PD-1 (Selleck), Vin (5 mg/kg) + isotype antibodies, Vin + anti–PD-1, rIL-24 (R&D Systems, Minneapolis, MN, USA), or rIL-24 + anti–PD-1. Vin was administrated orally once daily for 10 days, anti-PD-1 was given via intraperitoneal injection three times a week, and was injected intratumorally twice a week. Tumor growth was monitored every two days using caliper measurements, and the mice were euthanized once the tumor volume reached 1000 mm^3^. Tumor volumes (V) were calculated using the formula: V = [ width (mm) × length^2^ (mm^2^)] × 0.5.

To assess the role of ATF3 and IL-24 in Vin-induced antitumor effects, B16F10 cells stably transfected with shRNA targeting ATF3 or IL-24, along with corresponding non-targeting control cells, were subcutaneously injected into 8-week-old C57BL/6 female mice (5 × 10⁵ cells/mouse). Once the tumors reached 50–100 mm³, the mice were randomly divided into six groups: Mock + vehicle, Mock + Vin, shATF3 + vehicle, shATF3 + Vin, shIL-24 + vehicle, and shIL-24 + Vin treatment groups. Vin (10 mg/kg) was administered orally once daily for 10 days.

### Isolation of PBMCs from healthy donor blood samples

Peripheral blood mononuclear cells (PBMCs) were isolated from blood obtained from a healthy donor, who provided informed consent for the study, via Ficoll-Paque density gradient centrifugation. Ten milliliters of whole blood, collected in an EDTA tube, were diluted with an equal volume of PBS. In a 50 mL centrifuge tube, 10 mL of separation medium was added, and the diluted blood sample was carefully layered on top, ensuring a clear interface between the two liquids. The tube was centrifuged at 800 × g for 30 min at room temperature, with a gradual acceleration to facilitate the separation of blood components. PBMCs, located at the interface between the plasma and the separation medium, were carefully aspirated using a sterile pipette. The collected PBMCs were transferred to a new 50 mL tube and washed twice with PBS by centrifuging at 250 × g for 10 min, discarding the supernatant after each wash. The final PBMC pellet was resuspended in RPMI-1640 medium as required for subsequent experiments.

### Isolation of mouse PBMCs and CD8^+^ T cells

Mouse PBMCs were isolated from the spleens of 8-week-old C57BL/6 mice using a mouse lymphocyte isolation solution (DAKEWE, Shenzhen, Guangdong, China), following the manufacturer’s protocol. CD8^+^ T cells were subsequently purified from the PBMCs using the EasySep™ Mouse CD8^+^ T Cell Isolation Kit (STEMCELL, Shanghai, China), with a separation efficiency exceeding 95%, as verified by flow cytometry. The isolated CD8^+^ T cells were cultured in complete medium consisting of RPMI-1640, 10% FBS, 1% HEPES, 1% NEAA, 1% sodium pyruvate, 1% penicillin/streptomycin, 55 µM β-mercaptoethanol (β-ME), and 50 IU/mL recombinant mouse interleukin-2 (mIL-2). For activation, cells were treated with Dynabeads^®^ Mouse T-Activator CD3/CD28 (Thermo Fisher Scientific).

### Flow cytometry analysis

For analysis of the number and function of tumor-infiltrating immune cells, divest tumor tissue from euthanized mice and prepare a single-cell suspension. Divide each sample into two groups (innate immune cells and lymphocytes), and mix at least 10% of the samples with other samples for control, such as fluorescence subtraction one (FMO), single staining, and unstained groups. The samples were removed into a U-bottom 96-well plate and treated with Zombie Aqua^™^ dye (1:500, Biolegend, #423102, San Diego, CA, USA) for 15 min at room temperature in PBS followed by a wash with PBS. Then, the samples were incubated for 10 min at 4℃ with anti-CD16/CD32 to block Fc receptors (1:500, Biolegend, #101320). The samples were stained with surface markers antibody for 30 min at 4 °C in the dark, followed by washing with PBS. Subsequently, according to the manufacturer’s instructions, intracellular markers were stained with the eBioscience FOXP3 Transcription Factor Staining Buffer Set (Invitrogen, California USA). The following markers were used for immune cell characterization: CD3 for T cells; CD4 and CD8a for T cell subsets; MHC-II and F4/80 for M1 macrophages; CD206 and F4/80 for M2 macrophages; NK1.1 for NK cells; Foxp3 for regulatory T cells (Tregs); and CD45 to identify total leukocytes. Flow cytometry data were acquired on a 5 laser BD LSRFortessa™ X-20 system and analyzed using FlowJo 10.8.1 software (BD). The specific antibody clones and sources used for mouse and human cells are provided in Table S3.

A Transwell system was used to analyze the function of CD8^+^ T cells in PBMCs co-cultured with Vin-treated melanoma cells. Vin-treated melanoma cells were placed in the upper chamber, while PBMCs were seeded in the lower chamber. After 24 h of co-culturing, the PBMCs were harvested for staining and subsequent flow cytometry analysis.

### In vitro killing assays

In vitro tumor-killing assays were conducted using the Luciferase Assay System (Promega Corporation). The A375-luciferase and B16F10-luciferase melanoma cell lines were generated via lentiviral transduction and purified using fluorescence-activated cell sorting (BD FACSymphony™ S6, USA). The luciferase-expressing melanoma cells were seeded into 96-well black flat-bottom plates (Greiner, Pleidelsheim, Baden-Württemberg, Germany) at a density of 8 × 10^3^ cells per well, with or without Vin treatment. PBMCs were added to the wells at effector-to-target (E: T) ratios of 2.5:1 for human cells and 10:1 for mouse cells, or CD8^+^ T cells were added at an E: T ratios of 5:1 for mouse cells. The cells were then incubated for 24 h. After incubation, the supernatant was removed, and the cells were washed twice with PBS. Cells were lysed with 20 µL/well 1 × lysis buffer (Promega) on freeze-thaw cycle twice, then mixed with 20 µL/well luciferase reagent. Luminescence was measured using a BioTek Cytation 1 reader. Tumor cells without any treatment served as a negative control.

### Immunohistochemistry staining

After euthanizing mice bearing B16F10 tumor, tumor tissue was isolated, fixed with 4% paraformaldehyde, and subsequently embedded in paraffin. Immunohistochemistry staining was carried out according to the method described in reference [26]. Rabbit polyclonal Ki67 antibody (1:200, Servicebio, #GB111141, Wuhan, Hubei, China), and rabbit monoclonal IL-24 antibody (1:200, Proteintech, #26772) were used as primary antibodies and incubated overnight at 4 °C. We used an antibody diluent (ZSGB-BIO, Beijing, China) instead of the primary antibody as the negative control.

### Immunofluorescence staining

For in vitro staining, cells were seeded on sterile glass coverslips in 24-well plates and treated with Vin for 24 h. After PBS wash, cells were fixed with 4% paraformaldehyde for 10 min and permeabilized with 0.2% Triton X-100 for 5 min. After blocking with 3% BSA for 1 h, cells were incubated with anti-γH2AX antibody (1:100, CST, #9718s) overnight at 4 °C. After washing with PBST (0.1% Tween-20 in PBS), fluorophore-conjugated secondary antibodies (Alexa Fluro 488 donkey Anti-rabbit, Invitrogen) were applied for 1 h in the dark. Nuclei were counterstained with DAPI and coverslips were mounted with antifade mounting medium.

For tissue staining, formalin-fixed, paraffin-embedded tumor sections were deparaffinized in xylene and rehydrated through a graded ethanol series. Antigen retrieval was performed by heating sections in citrate buffer (pH 6.0) for 10 min. After cooling and washing, sections were blocked with 5% normal serum for 1 h at room temperature and incubated with anti-ATF3 antibody (1:100, Abcam, ab207434) overnight at 4 °C. After washing, sections were incubated with fluorophore-conjugated secondary antibodies for 1 h in the dark. Nuclei were counterstained with DAPI and sections were mounted using antifade mounting medium. Fluorescence images were captured using a fluorescence microscope.

### Statistical analysis

Statistical analysis was performed using GraphPad Prism 8.0 software. Data are expressed as mean ± standard deviation (SD), and comparisons between groups were made using either a t-test or an ordinary one-way ANOVA. A *p*-value of less than 0.05 was considered statistically significant in all experiments.

## Results

### Vin attenuates melanoma progression by reducing proliferation and invasiveness

**Fig. 1:**
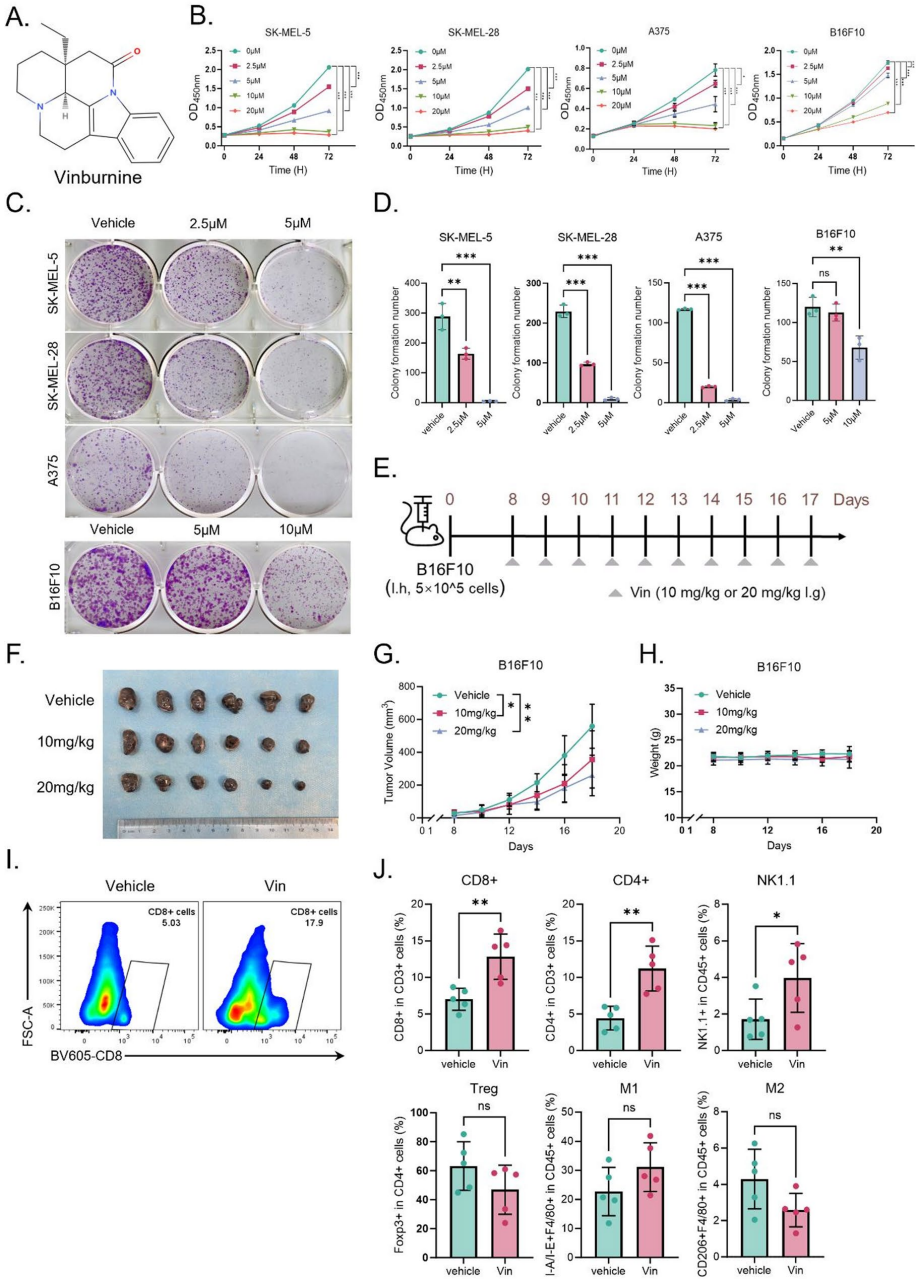
Vin inhibits melanoma progression. **A** Chemical structure of vinburnine. **B** The proliferation of melanoma cells (SK-MEL-5, SK-MEL-28, A375, and B16F10) was assessed by CCK-8 assay at 24, 48, and 72 h following treatment with Vin. *n* = 6. Data were presented as means ± SD, ***P < 0.001 according to one-way ANOVA. **C**,** D** Colony formation assay of SK-MEL-5, SK-MEL-28, and A375 cells treated with 2.5 and 5 µM of Vin for 48 h. Colony formation assay of B16F10 cells treated with 5 and 10 µM of Vin for 48 h. *n* = 3. Data were presented as means ± SD, ns., not significant, **P < 0.01,***P < 0.001 according to one-way ANOVA. **E** Schematic representation of the experimental design for animal treatment. **F**,** H** Tumor appearance, tumor volume, and body weight of mice treated with vehicle or Vin (10 mg/kg and 20 mg/kg). *n* = 6. Data were presented as means ± SD, *p < 0.05, **p < 0.01 according to one-way ANOVA. **I** Representative flow cytometric gating strategy for CD8^+^ T cells and quantification of CD8^+^ T cells. **J** Flow cytometric quantification of CD4^+^ T cells, NK cells, Treg cells, M1 macrophages, and M2 macrophages in tumor-bearing mice. *n* = 5. Data were presented as means ± SD, ns., not significant, *p < 0.05, **p < 0.01 according to paired t-test. Treg: regulatory T cells, M1: M1-type macrophages, M2: M2-type macrophages

To comprehensively investigate the role of Vin in melanoma progression, we performed both in vitro and in vivo experiments to assess its effects on cell proliferation, migration, invasion, and tumor growth. Vin is an alkaloid containing a benzene ring extracted from plants of the *Catharanthus genus* (Fig. 1A). It is widely used clinically to treat ischemic stroke and other cerebrovascular diseases. CCK-8 assays demonstrated that Vin significantly reduced the viability of human melanoma cell lines A375, SK-MEL-28, SK-MEL-5, and murine melanoma cell line B16F10 in a dose- and time-dependent manner, with IC50 values of approximately 5 µM for A375, SK-MEL-28, and SK-MEL-5, and around 7.5 µM for B16F10. CCK-8 assays demonstrated that Vin significantly reduced the viability of human melanoma cell lines SK-MEL-5, SK-MEL-28, A375, and murine melanoma cell line B16F10 in a dose- and time-dependent manner, with IC_50_ values of approximately 5 µM for SK-MEL-5, SK-MEL-28, and A375, and around 7.5µM for B16F10 (Fig. 1B, Supporting Information Fig. S1**A**). In contrast, Vin did not exhibit a cytotoxic effect on normal human melanocytes (PIG1), even at a high concentration of 20 µM (Fig. S1**B**). Colony formation assays further confirmed that Vin dose-dependently suppressed the colony-forming ability of SK-MEL-28, A375, SK-MEL-5, and B16F10 cells **(**Fig. 1C-D). Flow cytometry assessed cell cycle distribution to further explore Vin’s impact on cell proliferation. The data revealed that Vin treatment markedly arrested the G0/G1 phase of melanoma cells (Fig. S1**C-D**). In addition, both wound healing (Fig. S2A-C) and Transwell invasion assays (Fig. S2D-F) revealed that Vin markedly inhibited the migration and invasion of A375, SK-MEL-28, and SK-MEL-5 cells. These in vitro findings demonstrate that Vin effectively suppresses melanoma cell proliferation, migration, and invasion, prompting further investigation of its antitumor efficacy in vivo. To this end, B16F10 melanoma cells were subcutaneously inoculated into C57BL/6 mice, and tumor growth was monitored (Fig. 1E). Compared with the vehicle control group, Vin treatment (10 and 20 mg/kg) significantly inhibited tumor growth in B16F10 tumor-bearing mice (Fig. 1F-G). Notably, no significant changes in body weight were observed during Vin treatment (Fig. 1H), indicating that Vin exerts a potent antitumor effect in vivo without causing apparent systemic toxicity. To assess the effect of Vin (10 mg/kg) on the TME, we analyzed immune cell infiltration within the tumor tissues using flow cytometry. Vin treatment significantly enhanced the infiltration of CD8⁺ T cells, CD4⁺ T cells, and NK cells in the tumor compared to the control group (Fig. 1I-J). Additionally, in tumor tissues treated with Vin, we observed a slight reduction in the infiltration of regulatory T cells (Treg) and M2 macrophages, while M1 macrophage infiltration was slightly increased; however, these differences were not statistically significant (Fig. 1J, Fig. S3A-B). Together, these results suggest that Vin not only inhibits tumor growth but also enhances antitumor immunity by reshaping the immune landscape of the TME.

### Vin induces ROS overproduction, DNA damage, and apoptosis in melanoma cells

**Fig. 2:**
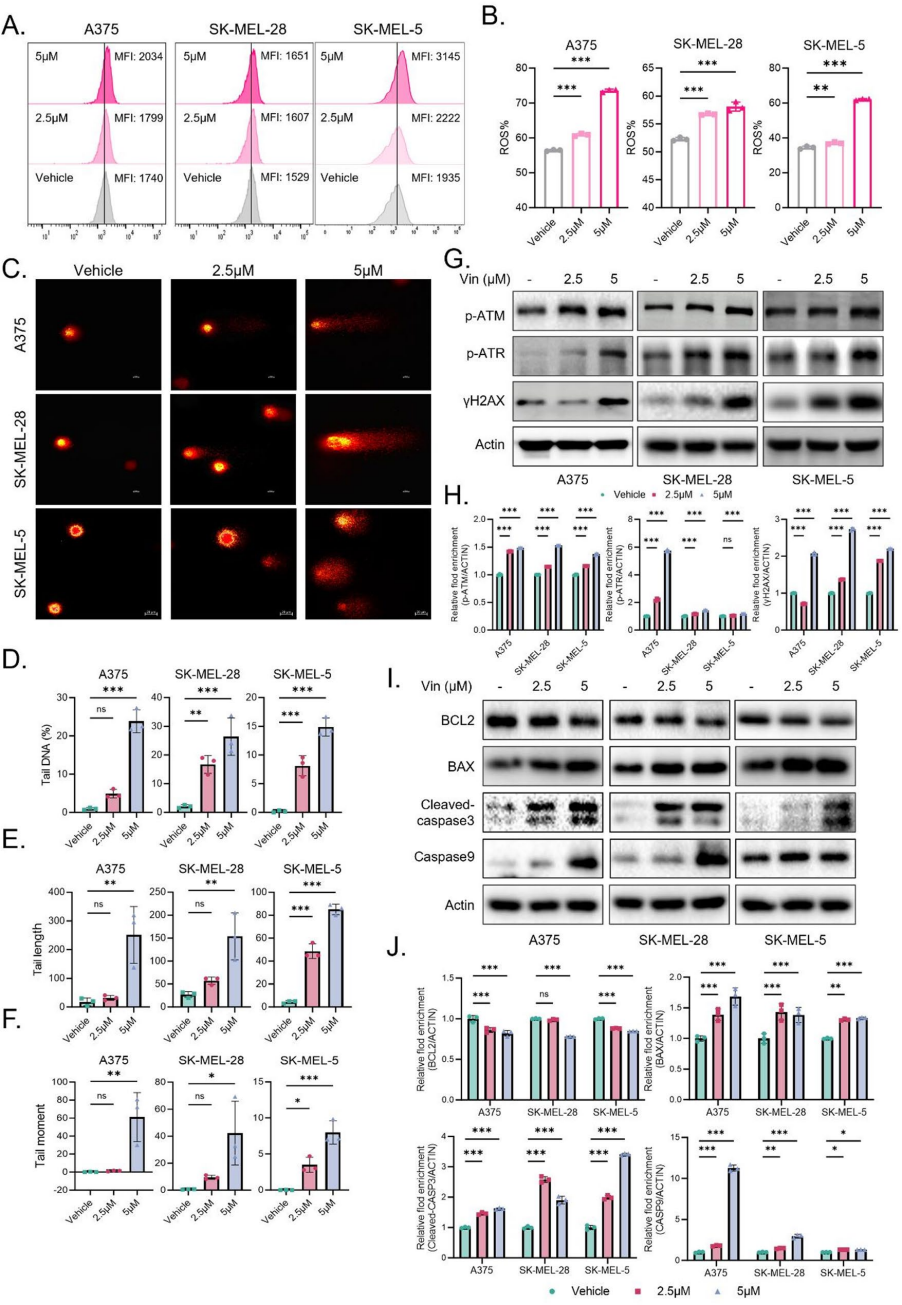
Effect of Vin on ROS generation, DNA damage, and apoptosis in melanoma cells. **A**,** B** Flow cytometric analysis of ROS levels in A375, SK-MEL-28, and SK-MEL-5 cells treated with 2.5 and 5 µM of Vin for 6 h. *n* = 3. Data were presented as means ± SD, ***p < 0.001 according to one-way ANOVA. **C** Comet assay assessing DNA damage in A375, SK-MEL-28, and SK-MEL-5 cells treated with 2.5 and 5 µM of Vin for 24 h. *n* = 3. Data were presented as means ± SD, ns., not significant, *p < 0.05, **p < 0.01, ***p < 0.001 according to two-way ANOVA. Quantification of comet assay data, including tail DNA **(D)**, tail length **(E)**, and tail moment **(F)**. *n* = 3. Data were presented as means ± SD, ns., not significant, *p < 0.05, **p < 0.01, ***p < 0.001 according to one-way ANOVA. **G**,** H** Western blot analysis of DNA damage markers, p-ATM, p-ATR, and γH2AX in A375, SK-MEL-28, and SK-MEL-5 cells treated with 2.5 and 5 µM of Vin for 24 h. *n* = 3. Data were presented as means ± SD, ns., not significant, ***p < 0.001 according to one-way ANOVA. **I**,** J** Western blot analysis of apoptosis markers, BCL2, BAX, cleaved-caspase3, and caspase9 in A375, SK-MEL-28, and SK-MEL-5 cells treated with 2.5 and 5 µM of Vin for 48 h. *n* = 3. Data were presented as means ± SD, ns., not significant, *p < 0.05, **p < 0.01, ***p < 0.001 according to one-way ANOVA

To explore whether the anti-melanoma effects of Vin are mediated by ROS accumulation, we first measured intracellular ROS levels in melanoma cells following Vin treatment using flow cytometry. As shown in Fig. 2A-B, Vin markedly elevated ROS production. To verify the functional significance of ROS accumulation, we co-treated the cells with Vin and the ROS scavenger NAC. NAC effectively reversed the Vin-induced growth inhibition of melanoma cells (Fig. S4A), suggesting that ROS generation is a critical mediator of Vin’s antitumor activity. Given that elevated ROS levels are known to induce oxidative DNA damage and disrupt genome integrity [27], we next examined whether Vin provokes DNA damage. Comet assays revealed pronounced DNA strand breaks in Vin-treated cells, as evidenced by increased tail length, tail DNA content, and tail moment compared to controls (Fig. 2C-F). To further explore the DNA damage response (DDR) pathway was activated, we analyzed canonical DDR markers including p-ATM, p-ATR, and γH2AX. Western blot analysis showed robust upregulation of these proteins in A375, SK-MEL-28, SK-MEL-5, and B16F10 cells after Vin exposure (Fig. 2G-H, Fig. S4B-C). Consistently, immunofluorescence staining revealed a notable increase in γH2AX-positive cells upon Vin treatment in A375 and SK-MEL-28 cells (Fig. S4D-E). Since ROS-induced DNA damage is often coupled with apoptosis [28], we next evaluated cell death in response to Vin. Flow cytometry demonstrated a significant rise in early apoptotic populations in Vin-treated cells (Fig. S4F-G). At the molecular level, Vin increased the expression of pro-apoptotic proteins, such as BAX, cleaved caspase-3, and cleaved caspase-9, while downregulating the anti-apoptotic factor BCL-2 (Fig. 2I-J). Together, these results suggest that Vin exerts its antitumor effects by inducing oxidative stress, leading to DNA damage, DDR activation, and apoptosis in melanoma cells.

### Vin significantly upregulates IL-24 expression in melanoma cells

**Fig. 3:**
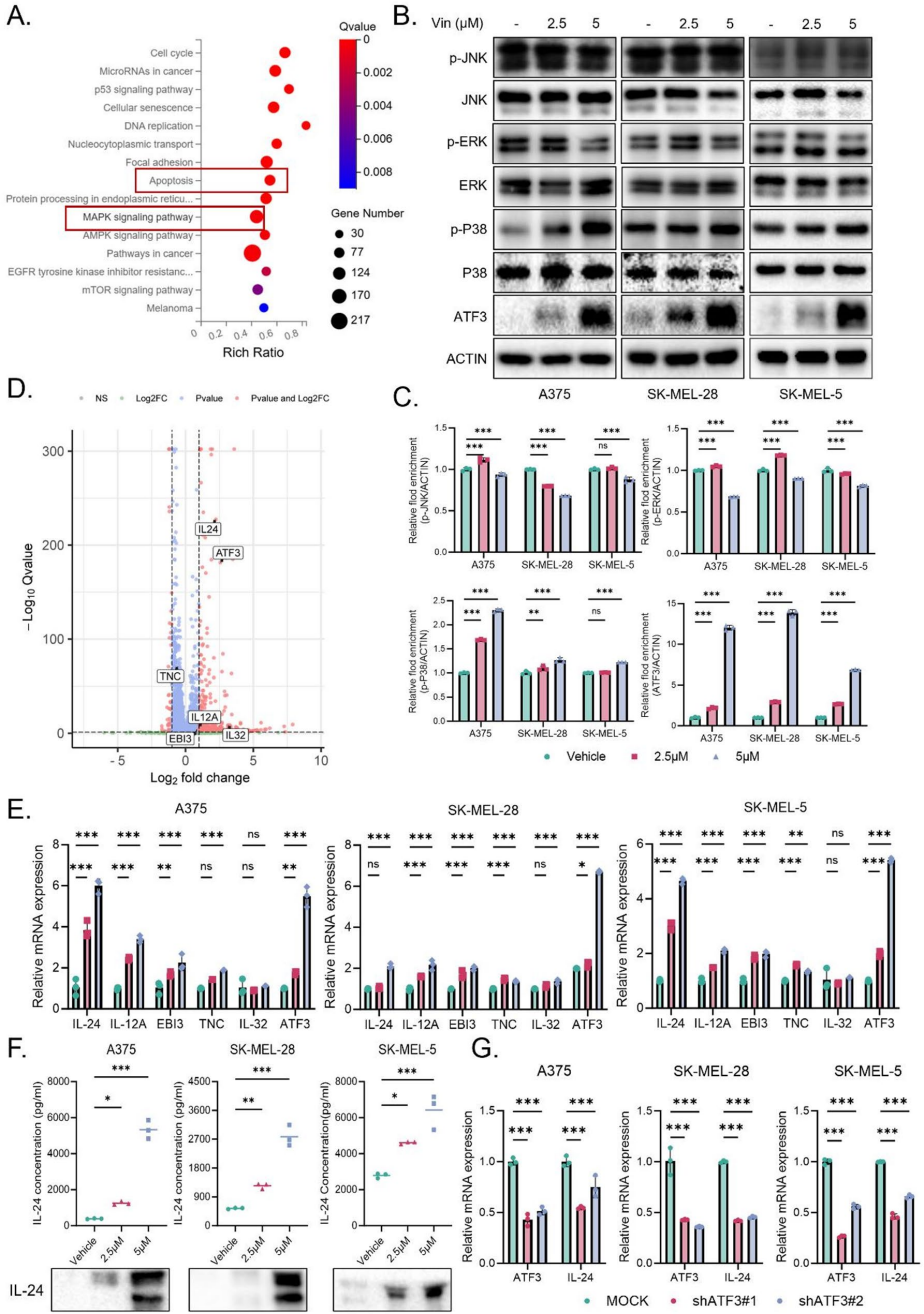
Molecular profiling and functional validation of Vin treatment in melanoma cells. **A** KEGG pathway enrichment analysis of the differentially expressed genes. **B**,** C** Western blot analysis of MAPK signaling pathway markers and the transcription factor ATF3 expression in melanoma cells treated with 2.5 or 5 µM of Vin for 48 h. *n* = 3. Data were presented as means ± SD, ns., not significant, *p < 0.05, **p < 0.01, ***p < 0.001 according to one-way ANOVA. **D** Volcano plot of DEGs from RNA sequencing, with cytokines and ATF3 highlighted. **E** RT-PCR validation of differentially expressed cytokines (IL24, IL12A, EBI3, TNC, ATF3) in A375, SK-MEL-28, and SK-MEL-5 cells based on RNA sequencing data. *n* = 3. Data were presented as means ± SD, ns., not significant,*p < 0.05, **p < 0.01,***p < 0.001 according to one-way ANOVA. **F** ELISA and western blot measurement of IL-24 levels in the conditioned medium from A375, SK-MEL-28, and SK-MEL-5 cells treated with 2.5 or 5 µM of Vin for 48 h. *n* = 3. Data were presented as means ± SD, *p < 0.05, **p < 0.01, ***p < 0.001 according to one-way ANOVA. **G** RT-PCR analysis of ATF3 and IL-24 mRNA expression in A375 and SK-MEL-28 cells with ATF3 knockdown. *n* = 3. Data were presented as means ± SD, ***p < 0.001 according to two-way ANOVA. Ctrl: control, IL-24: interleukin 24, IL-12 A, interleukin 12 A, IL-32, interleukin 32, EBI3: Epstein-barr virus induced 3, TNC: Thrombospondin 1, ATF3: activating transcription factor 3

To further investigate the potential mechanisms underlying Vin’s inhibitory effect on melanoma cell growth, we performed transcriptomic analysis on A375 cells, comparing gene expression profiles among the high-dose, low-dose, and solvent control groups. The results showed 818 upregulated and 1177 downregulated genes in the low-dose group, and 2702 upregulated and 3122 downregulated genes in the high-dose group, with 1846 differentially expressed genes (DEGs) common to both treatment groups (Fig. S5A). KEGG pathway enrichment analysis revealed that Vin treatment significantly affected multiple signaling pathways. In the high-dose group, pathways related to the cell cycle, DNA replication, and apoptosis were notably enriched **(**Fig. 3A**)**. Notably, MAPK signaling was also among the enriched pathways. Consistent with this finding, Western blot analysis demonstrated that Vin selectively enhanced the phosphorylation of P38, with negligible effects on p-JNK and p-ERK, suggesting a preferential activation of the P38/MAPK axis (Fig. 3B-C, Fig. S5D-E). Given our previous findings that Vin treatment enhanced CD8⁺ T cell infiltration in the tumor microenvironment, we subsequently interrogated the transcriptomic data to identify differentially expressed cytokines that might contribute to T cell-mediated cytotoxicity. Volcano plot analysis showed that Vin treatment significantly upregulated IL-24, IL-12 A, IL-32, TNC, and EBI3 (Fig. 3D). RT-PCR validation confirmed that IL-24 expression increased in both time- and dose-dependent manners, with the most notable changes observed in A375, SK-MEL-28, and SK-MEL-5 cells (Fig. 3E). Additionally, treatment with 10 µM Vin led to a significant increase in IL-24 mRNA levels in B16F10 cells (Fig. S5F). To further evaluate IL-24 protein expression, we performed ELISA and Western blot analyses on the culture supernatants of A375, SK-MEL-28, and SK-MEL-5 cells, which consistently demonstrated that Vin treatment markedly enhanced IL-24 secretion (Fig. 3F). For B16F10 cells, IL-24 protein expression in the supernatant was assessed by Western blot and showed a similar increase following Vin treatment (Fig. S5G). These findings suggest that IL-24 is a key effector molecule contributing to the anti-melanoma effects of Vin.

To explore the potential regulatory factors responsible for Vin-induced upregulation of IL-24, we focused on ATF3, a well-known downstream effector of the P38/MAPK pathway involved in cellular stress responses [29]. Our RNA-seq data showed a marked increase in ATF3 expression following Vin treatment (Fig. 3D). This finding was further validated by western blot and RT-PCR, which demonstrated strong induction of ATF3 in A375, SK-MEL-28, and SK-MEL-5 cells (Fig. 3B-C, E). Similarly, treatment with Vin also increased ATF3 mRNA and protein levels in B16F10 cells (Fig. S5D-F). Importantly, RT-PCR analysis revealed that ATF3 knockdown significantly decreased IL-24 mRNA levels in A375, SK-MEL-28, and SK-MEL-5 cells (Fig. 3G), suggesting that ATF3 is required for Vin-induced IL-24 transcription. Together, these results indicate that ATF3 may act as a key transcriptional regulator linking P38 activation to IL-24 upregulation, thereby contributing to the anti-melanoma effects of Vin.

### Vin upregulates IL-24 expression via the P38/MAPK/ATF3 pathway and enhances antitumor immunity in vivo

**Fig. 4:**
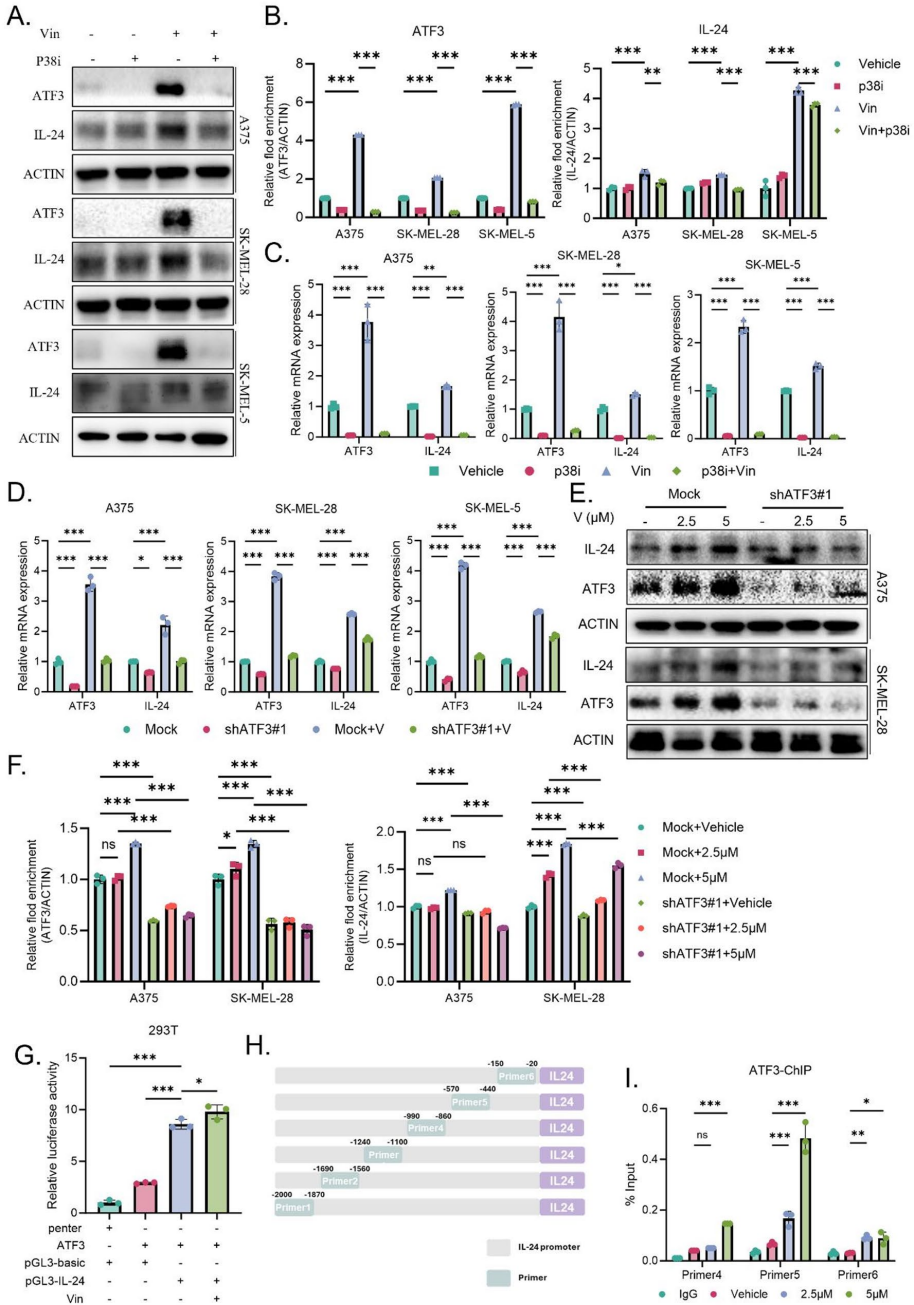
Vin regulated expression of IL-24 in melanoma cells by P38/MAPK/ATF3. **A**,** B** Western blot analysis of ATF3 and IL-24 protein expression in A375, SK-MEL-28, and SK-MEL-5 cells treated with Vin (5 µM) and P38 inhibitor SB203580 (P38i, 10 μM). *n* = 3. Data were presented as means ± SD, ns., not significant,**p < 0.01, ***p < 0.001 according to one-way ANOVA. **C** RT-PCR analysis of ATF3 and IL-24 mRNA levels in A375, SK-MEL-28, and SK-MEL-5 cells treated with Vin and P38i. *n* = 3. Data were presented as means ± SD, *p < 0.05, ***p < 0.001 according to two-way ANOVA. **D** RT-PCR analysis of ATF3 and IL-24 mRNA levels in ATF3 knockdown melanoma cells treated with Vin (5µM). *n* = 3. Data were presented as means ± SD, *p < 0.05, ***p < 0.001 according to two-way ANOVA. **E**,** F** Western blot analysis of ATF3 and IL-24 protein levels in ATF3 knockdown melanoma cells treated with Vin (5µM). *n* = 3. Data were presented as means ± SD, ns., not significant, ***p < 0.001 according to one-way ANOVA. **G** The dual-luciferase reporter assay showed binding of ATF3 to the IL-24 promoter upon Vin treatment. *n* = 3. Data were presented as means ± SD, *p < 0.05, ***p < 0.001 according to two-way ANOVA. **H** Schematic diagram of ChIP primers targeting the IL-24 promoter. **I** ChIP analysis showing ATF3 binding to the IL-24 promoter in SK-MEL-28 cells. *n* = 3. Data were presented as means ± SD, ns., not significant,*p < 0.05, **p < 0.01, ***p < 0.001 according to two-way ANOVA. IL-24: interleukin 24, ATF3: activating transcription factor 3, p38i, p38 inhibitor

To determine whether Vin upregulates IL-24 expression through the P38/MAPK/ATF3 signaling pathway, we treated melanoma cells with the P38 inhibitor SB203580 in combination with Vin. The results showed that SB203580 effectively suppressed both ATF3 and IL-24 mRNA and protein levels induced by Vin (**Fig**,** 4 A-C**), suggesting that activation of the P38/MAPK pathway is critical for upregulating ATF3 and IL-24. Furthermore, knockdown of ATF3 in A375, SK-MEL-28, and B16F10 cells significantly attenuated the Vin-induced increase in IL-24 expression (Fig. 4E-F, Fig. S5I-J), supporting a regulatory role for ATF3 in mediating IL-24 transcription. To further investigate whether ATF3 directly regulates IL-24 expression, we constructed a dual-luciferase reporter plasmid containing the IL-24 promoter region and performed luciferase assays. The results showed that ATF3 directly binds to the IL-24 promoter region, with Vin treatment enhancing this binding (Fig. 4G). ChIP assays confirmed the interaction between ATF3 and the IL-24 promoter (Fig. 4H-I). Together, these findings reveal a novel molecular mechanism through which Vin upregulates IL-24 expression by activating the P38/MAPK/ATF3 signaling pathway.

To validate the functional significance of the P38/MAPK/ATF3/IL-24 axis in vivo, we established melanoma xenograft models with stable knockdown of ATF3 or IL-24. Western blot analysis confirmed efficient knockdown of ATF3 and IL-24 in tumor cells prior to implantation (Fig. S6A). Upon Vin treatment, control tumors exhibited significant growth inhibition, whereas tumors with ATF3 or IL-24 knockdown showed markedly reduced sensitivity to Vin, with minimal suppression of tumor growth observed (Fig. S6B-C). No significant differences in mouse body weight were observed among the groups during treatment (Fig. S6D). Furthermore, flow cytometric analysis of tumor-infiltrating lymphocytes revealed that Vin-induced increases in CD8⁺ T cell infiltration, as well as the proportions of GZMB⁺ and IFNγ⁺ CD8⁺ T cells, were abolished in both ATF3- and IL-24-deficient tumors (Fig. S6E). These results demonstrate that the ATF3-IL-24 axis is essential for Vin-mediated enhancement of antitumor immunity and tumor growth inhibition in vivo.

### Vin enhances T cell-mediated cytotoxicity via IL-24/IL-20R2 signaling axis

**Fig. 5:**
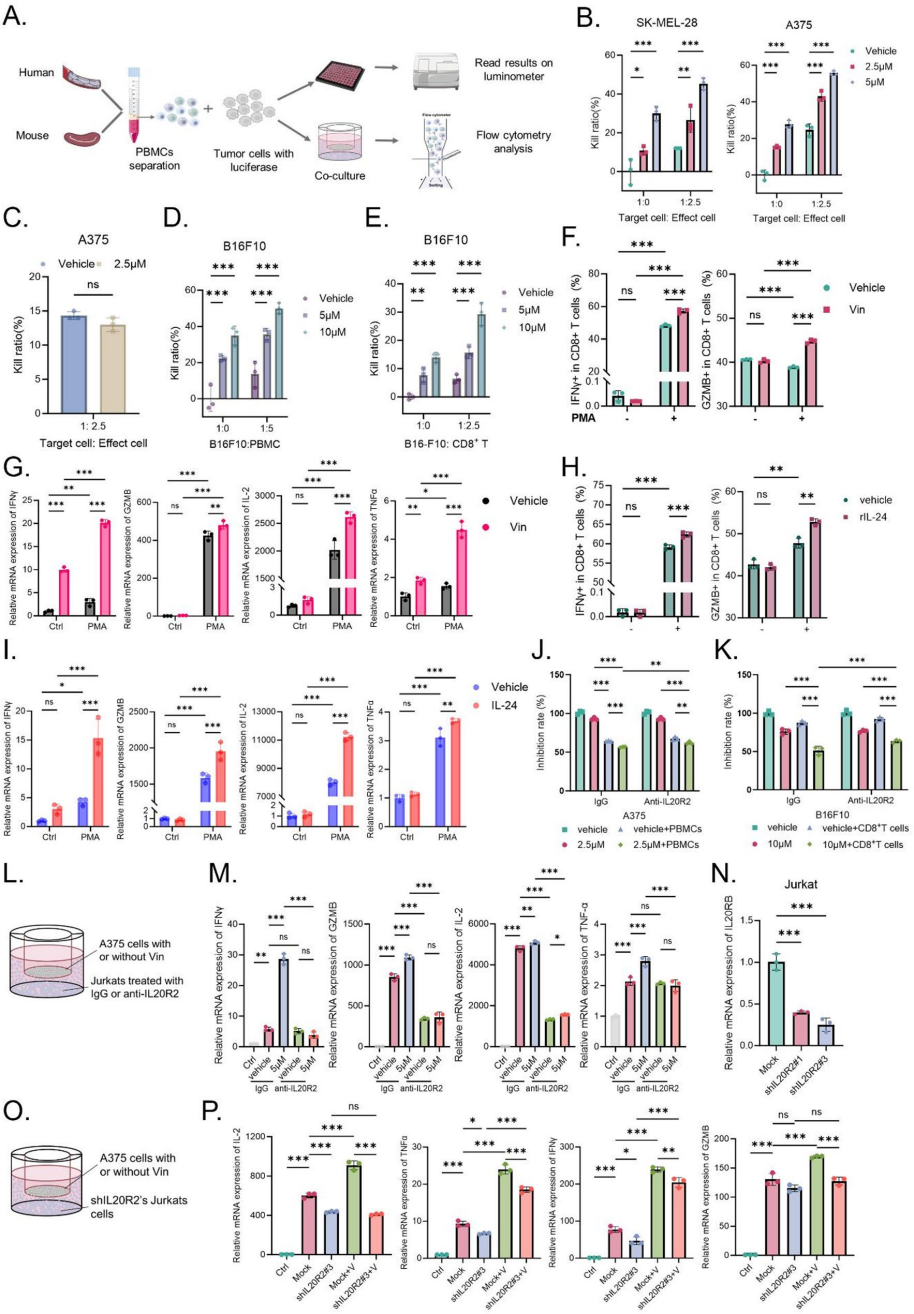
Vin enhances CD8^+^ T cell cytotoxicity by promoting IL-24 secretion in melanoma. **A** Schematic of human blood samples and mouse spleen tissue isolation of PBMCs for subsequent assays. **B** Luciferase-based cytotoxicity assay of Vin-treated luci-human melanoma cells co-cultured with or without 2.5× H-PBMCs for 12 h. *n* = 3. Data were presented as means ± SD, *p < 0.05, **p < 0.01, ***p < 0.001 according to two-way ANOVA. **C** Cytotoxicity assay of PBMCs pre-treated with Vin for 24 h, followed by co-culture with luci-A375 cells in a 2.5:1 ratio for 12 h, measured by luciferase detection. *n* = 3. Data were presented as means ± SD, ns., not significant according to paired t-test. **D** Luciferase-based cytotoxicity assay of Vin-treated luci-B16F10 cells with or without 5× M-PBMCs co-treatment for 24 h. *n* = 3. Data were presented as means ± SD, ***p < 0.001 according to two-way ANOVA. **E** Cytotoxicity assay of Vin-treated luci-B16F10 cells co-cultured with or without 2.5× activated CD8^+^ T cells for 12 h, detected by luciferase assay. *n* = 3. Data were presented as means ± SD,**p < 0.01, ***p < 0.001 according to two-way ANOVA. **F** Co-culture setup: the upper chamber contains tumor cells with or without Vin treatment, and the lower chamber contains H-PBMCs with or without activation. Flow cytometric analysis of functional markers (GZMB and IFNγ) in CD8^+^ T cells from the lower chamber of the co-culture system. *n* = 3. Data were presented as means ± SD, ns., not significant, ***p < 0.001 according to two-way ANOVA. **G** Co-culture setup: the upper chamber contains tumor cells with or without Vin treatment, and the lower chamber contains Jurkat cells with or without activation. RT-PCR analysis of IFNγ, GZMB, IL-2, and TNF-α expression in Jurkat cells from the co-culture system. *n* = 3. Data were presented as means ± SD, ns., not significant, *p < 0.05, **p < 0.01, ***p < 0.001 according to two-way ANOVA. **H** Flow cytometric analysis of GZMB and IFNγ in CD8^+^ T cells from H-PBMCs treated with rIL-24. *n* = 3. Data were presented as means ± SD, ns., not significant, **p < 0.01, ***p < 0.001 according to two-way ANOVA. **I** RT-PCR analysis of IFNγ, GZMB, IL-2, and TNF-α expression in Jurkat cells treated with rIL-24. *n* = 3. Data were presented as means ± SD, ns., not significant,*p < 0.05,**p < 0.01,***p < 0.001 according to two-way ANOVA. **J** Cytotoxicity assay of Luci-A375 cells treated with Vin, performed using the co-culture system described in (**A)**. Cells were co-cultured with H-PBMCs with or without anti-IL-20R2 for 12 h. *n* = 3. Data were presented as means ± SD, **p < 0.01,***p < 0.001 according to two-way ANOVA. **K** Cytotoxicity assay of Luci-B16F10 cells treated with Vin, performed using the co-culture system described in **(A)**. Cells co-cultured with CD8^+^ with or without anti-IL-20R2 for 24 h. *n* = 3. Data were presented as means ± SD, ***p < 0.001 according to two-way ANOVA. **L** Schematic illustration of the Transwell co-culture system: Vin-treated A375 cells were seeded in the upper chamber, while Jurkat cells treated with or without anti-IL-20R2 were placed in the lower chamber. **M** RT-PCR analysis of IFNγ, GZMB, IL-2, and TNF-α expression in Jurkat cells from the lower chamber of the Transwell co-culture system **(L)**. *n* = 3. Data were presented as means ± SD, ns., not significant,*p < 0.05,**p < 0.01,***p < 0.001 according to two-way ANOVA. **N** Knockdown Jurkat cells using two slow virus sequences IL-20R2 and detect mRNA levels by RT-PCR. *n* = 3. Data were presented as means ± SD, ns., not significant,*p < 0.05,**p < 0.01,***p < 0.001 according to two-way ANOVA. **O** Schematic illustration of the Transwell co-culture system: Vin-treated A375 cells were seeded in the upper chamber, while IL-20R2-knockdown Jurkat cells were placed in the lower chamber. **P** RT-PCR analysis of IFNγ, GZMB, IL-2, and TNF-α expression in Jurkat cells from the lower chamber of the Transwell co-culture system **(O)**. *n* = 3. Data were presented as means ± SD,***p < 0.001 according to two-way ANOVA. PBMCs: peripheral blood mononuclear cell, PMA: Phorbol 12-myristate 13-acetate, rIL-24: recombinant interleukin 24

Building on previous in vivo observations suggesting that CD8⁺ T cells play a pivotal role in Vin-induced antitumor immunity, we established an in vitro co-culture system to explore how Vin activates CD8⁺ T cells to mediate antitumor immune responses. PBMCs were isolated from healthy donors and mouse spleens, then co-cultured with luciferase-expressing melanoma cells in the presence of Vin to assess the cytotoxic effects of PBMCs on melanoma cells. Using a luciferase reporter system, we measured melanoma cell activity in the co-treatment model involving Vin, PBMCs, and melanoma cells (Fig. 5A). The results demonstrated that Vin significantly enhanced PBMC-mediated cytotoxicity against SK-MEL-28 and A375 cells in a dose-dependent manner (Fig. 5B). When PBMCs were pre-treated with Vin before co-incubation with melanoma cells, no increase in PBMC-mediated cytotoxicity was observed compared to the control group (Fig. 5C). These findings suggest that Vin acts on melanoma cells to induce the secretion of immunomodulatory factors, rather than directly enhancing T cell function. To further investigate the effect of Vin on immune cell-mediated cytotoxicity, we employed a co-culture model involving mouse-derived PBMCs, B16F10 cells, and Vin. Similar to the results with human melanoma cells, Vin treatment significantly enhanced PBMC-mediated killing of B16F10 cells (Fig. 5D). We further isolated and activated CD8⁺ T cells mouse spleens using magnetic bead sorting and co-cultured them with B16F10 cells. Consistent with the PBMC results, Vin significantly enhanced the cytotoxicity activity of CD8⁺ T cells against B16F10 cells (Fig. 5E, Fig. S7A). Next, we examined changes in functional markers in CD8⁺ T cells and Jurkat cells within the co-culture system using flow cytometry and RT-PCR. Flow cytometric analysis revealed that in the activated state, the proportion of IFNγ^+^CD8⁺ T cells and GZMB^+^CD8⁺ T cells was significantly increased in PBMCs co-cultured with Vin-treated melanoma cells (Fig. 5F). Additionally, RT-PCR analysis showed that Jurkat cells co-cultured with Vin-treated melanoma cells exhibited significantly higher mRNA levels of IFNγ, GZMB, IL-2, and TNFα in the activated state. The upregulation of IL-2 and TNFα confirmed the successful activation of Jurkat cells (Fig. 5G). To further explore the role of IL-24 in this process, we collected conditioned medium (CM) from either Vin-treated or untreated melanoma cells and used it to treat Jurkat cells. The results demonstrated that, in the activated state, CM from Vin-treated melanoma cells similarly enhanced the expression of IFNγ, GZMB, IL-2, and TNFα in Jurkat cells (Fig. S7B). Given the increased IL-24 secretion by Vin-treated melanoma cells, we hypothesized that Vin enhances CD8⁺ T cell function through IL-24 secretion from melanoma cells. To test this hypothesis, we stimulated PBMCs and Jurkat cells with recombinant IL-24 (rIL-24) protein and analyzed functional markers using flow cytometry and RT-PCR. The results showed that rIL-24 treatment significantly increased the proportion of IFNγ^+^CD8⁺ T cells and GZMB^+^CD8⁺ T cells in PBMCs (Fig. 5H), and enhanced the expression of IFNγ, GZMB, IL-2, and TNFα in Jurkat cells (Fig. 5I).

To further investigate the mechanism by which Vin modulates CD8⁺ T cell-mediated antitumor immunity, we intervened in the IL-24/IL-20R2 signaling pathway to assess whether it plays a critical role in Vin-induced effects. First, we added an anti-IL-20R2 antibody (anti-IL-20R2) or isotype control IgG to the co-culture system to assess whether anti-IL-20R2 affects the Vin-induced enhancement of T cell-mediated cytotoxicity. The results showed that compared to the isotype IgG control, anti-IL-20R2 significantly reduced the enhanced cytotoxicity of PBMCs against A375 cells and CD8⁺ T cells against B16F10 cells, both induced by Vin (Fig. 5J-K). Additionally, anti-IL-20R2 treatment reduced the expression of IFNγ, GZMB, IL-2, and TNFα in Jurkat cells co-cultured with Vin-treated melanoma cells (Fig. 5L-M). Next, we used flow sorting to establish stable IL-20R2 knockdown Jurkat cells, which were then co-cultured with Vin-treated melanoma cells (Fig. 5N, Fig. S7C). Knockdown of IL-20R2 significantly suppressed the upregulation of IFNγ, GZMB, IL-2, and TNFα in Jurkat cells activated by co-culture and Vin treatment (Fig. 5O-P). Additionally, we constructed IL-24-silenced luci-A375 cells and co-incubated them with Vin and PBMCs to assess PBMC-mediated cytotoxicity against the IL-24-silenced melanoma cells. The results indicated that silencing IL-24 notably reversed the suppressive effect of Vin combined with PBMCs on A375 cells (Fig. S7D-F). Furthermore, flow cytometry analysis revealed a significant decrease in the proportion of IFNγ^+^CD8^+^ T cells and GZMB^+^CD8^+^ T cells in the IL-24-silenced A375 group within the co-culture system (Fig. S7G). Together, these findings confirm that Vin promotes IL-24 secretion from melanoma cells, activating the IL-20R2 signaling axis to enhance CD8⁺ T cell cytotoxicity and amplifying antitumor immune responses. These results offer new insights into the mechanisms underlying Vin’s antitumor effects and highlight the IL-24/IL-20R2 signaling axis as a promising target for immunotherapy.

### Vin combined with PD-1 Blockade effectively inhibits melanoma growth in vivo

**Fig. 6:**
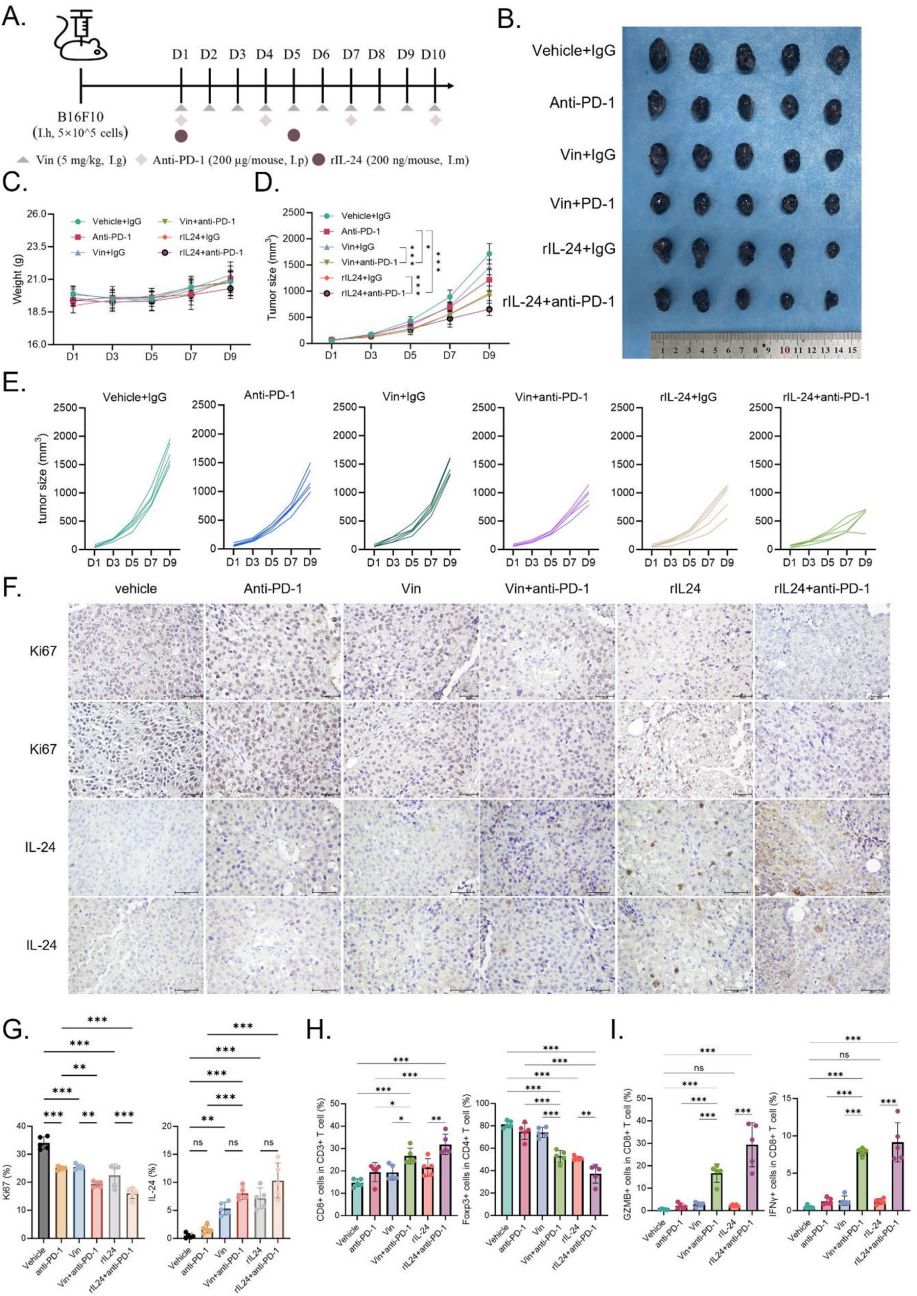
The combination of Vin and PD-1 blockage suppresses melanoma growth. **A** Schematic of treatment regimen in C57BL/6 mice bearing B16F10 tumors, including PBS, anti-PD-1, Vin, Vin + anti-PD-1, rIL-24, and rIL-24 + anti-PD-1 groups. **B** Representative images of dissected B16F10 tumors from treated mice. **C** Mouse body weight was measured during the treatment period. **D**,** E** Tumor growth curves of B16F10 tumors in C57BL/6 immune-competent mice treated with indicated treatments. *n* = 5. Data were presented as means ± SD,*p < 0.05,***p < 0.001 according to one-way ANOVA. **F** Immunohistochemical staining of Ki67 and IL-24 in tumor tissues. Scale bars, 100 μm. Statistical analysis of IHC staining is shown in (**G**). *n* = 5. Data were presented as means ± SD, ns., not significant,**p < 0.01,***p < 0.001 according to one-way ANOVA. **H** Flow cytometry analysis of CD8^+^ T cells, GZMB^+^CD8^+^ T cells, IFNγ^+^CD8^+^ T cells, and Treg cells in tumor tissue. *n* = 5. Data were presented as means ± SD, ns., not significant,**p < 0.01,***p < 0.001 according to one-way ANOVA. I.g.: Intragastric administration, I.p.: Intraperitoneal injection, I.m.: Intramuscular injection

Combining immune checkpoint inhibitors with other therapeutic approaches holds promise for improving melanoma treatment outcomes. To assess the therapeutic potential of Vin in conjunction with an immune checkpoint inhibitor, we investigated the effects of an anti-PD-1 antibody combined with Vin in a B16F10 mouse tumor model (Fig. 6A). The results indicated that, compared to the control group or monotherapies with either Vin or anti-PD-1 antibody, the combination treatment significantly enhanced tumor suppression. This was evident from a reduced tumor growth rate and significantly smaller tumor volumes (Fig. 6B-E). Importantly, no notable changes in body weight or obvious signs of toxicity were observed, supporting the safety profile of the combined therapy (Fig. 6D). To validate further the role of IL-24 in mediating Vin’s antitumor effects, we compared the efficacy of rIL-24 alone and in combination with anti-PD-1 antibody. The combination of rIL-24 and anti-PD-1 significantly enhanced the antitumor efficacy of rIL-24, indicating a synergistic effect between the two (Fig. 6B-E). Both immunohistochemistry and ELISA confirmed the effective delivery of rIL-24 within the tumor tissue (Fig. 6F, Fig. S8A). Moreover, IL-24 expression was markedly upregulated in tumors following Vin treatment, as shown by immunohistochemical staining **(**Fig. 6F**)**, further supporting its role as a downstream effector of Vin-induced signaling. Histological analysis revealed that, compared to monotherapies with Vin, anti-PD-1, or rIL-24, the combination treatments significantly reduced Ki67 expression, indicating more efficient inhibition of tumor proliferation (Fig. 6F-G). In line with the proposed mechanism, immunofluorescence analysis demonstrated a marked upregulation of ATF3 expression in tumor tissues from Vin-treated mice (Fig. S8B), supporting the in vivo activation of the P38/MAPK/ATF3 signaling pathway and its role in mediating IL-24 expression. Additionally, flow cytometry analysis revealed that the combination therapies, in contrast to the individual treatments, significantly increased CD8⁺ T cell infiltration into the TME while decreasing Treg cell infiltration (Fig. 6H, Fig. S8C). Furthermore, IFNγ and GZMB expression in CD8⁺ T cells was markedly elevated in the combination groups (Fig. 6I). These findings suggest that Vin enhances the efficacy of PD-1 blockade therapy by modulating the tumor immune microenvironment and boosting antitumor immune responses.

**Fig. 7:**
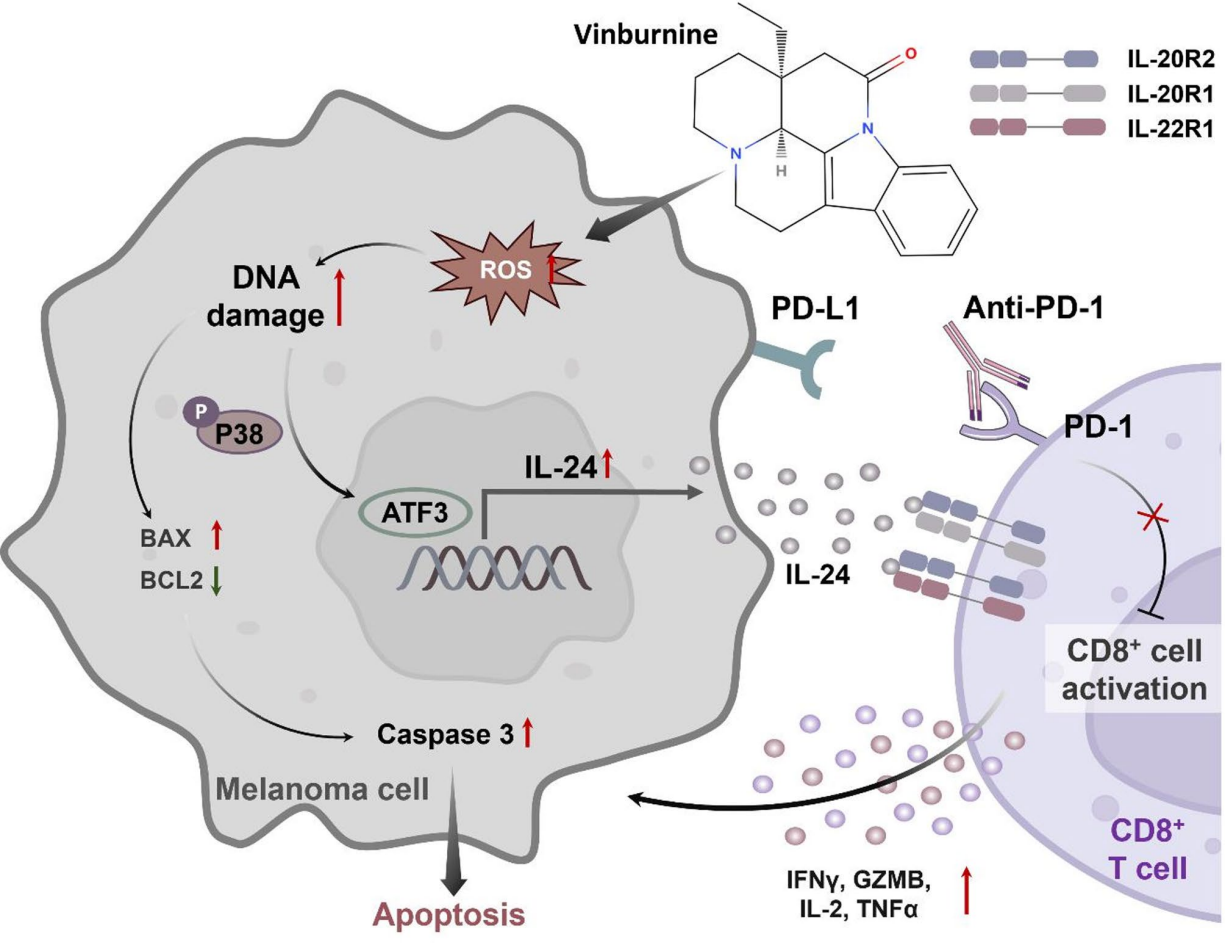
Vinburnine induces melanoma cell apoptosis via ROS and DNA damage, and promotes IL-24 secretion through the P38/MAPK/ATF3 pathway, activating CD8^+^ T cells. Its combination with PD-1 blockers enhances antitumor immunity

### High expression of IL-24 in melanoma is positively associated with antitumor immunity

To further explore the correlation between IL-24 expression in melanoma tumors, CD8^+^ T cell infiltration, the response to anti-PD-1 therapy, and its potential impact on patient prognosis, we first analyzed data from the public database. EPIC scoring of TCGA melanoma data revealed that the high IL-24 expression group showed a significant increase in B cell and CD8^+^ T cell infiltration compared to the low expression group. No significant differences were observed in the infiltration of endothelial cells, CD4^+^ T cells, macrophages, or NK cells (Fig. S9A). Next, we examined IL-24 expression across different cell types using the SKCM_GSE72056 single-cell dataset. IL-24 was highly expressed in B cells but showed low expression in malignant and endothelial cells (Fig. S9B). Interestingly, while IL-24 expression did not correlate with overall survival in melanoma patients (Fig. S9C), it was associated with improved survival outcomes in patients treated with anti-PD-1 therapy (Fig. S9D). Further analysis indicated that IL-24 levels were significantly higher in melanoma patients who responded to anti-PD-1 therapy, although its expression did not vary substantially before and after treatment (Fig. S9E-F). This suggests that anti-PD-1 therapy may not directly regulate IL-24 expression. Taken together, these findings highlight a strong association between high IL-24 expression and enhanced antitumor immune responses in melanoma. Moreover, IL-24 may play a role in modulating the effectiveness of anti-PD-1 therapy and influencing patient prognosis.

## Discussion

Natural products have consistently served as a valuable source of innovation in cancer research and drug development [30]. Previous studies have shown that vinca alkaloids exhibit potent antitumor activity. These compounds exert their effects by binding to microtubules, inhibiting microtubule polymerization and disrupting the cell division process, which ultimately suppresses tumor cell proliferation [31, 32]. Due to their strong cytotoxicity, vinca alkaloids have been widely used in chemotherapy for various cancers, demonstrating promising therapeutic efficacy, particularly in the treatment of Hodgkin’s lymphoma [33], ovarian cancer [34], and small cell lung cancer [35]. Vinburnine, a metabolite of vinca alkaloids and vindesine, is used in the treatment of cerebrovascular diseases, including ischemia and hypoxia [36, 37]. Previous studies have reported that vinburnine exhibits selective antitumor activity across different cancer types [38, 39]. However, there is limited research on its application in melanoma. Our findings demonstrate that vinburnine triggers ROS generation, resulting in DNA damage and the activation of apoptosis, highlighting its therapeutic potential in melanoma treatment.

ROS are key players in melanoma cells, where they induce DNA double-strand breaks (DSBs) [39–41]. As highly reactive molecules, ROS can directly or indirectly cause DNA damage, leading to oxidative stress and genomic instability. Under normal conditions, ROS levels are tightly regulated, but in tumor cells, due to metabolic reprogramming and high proliferation rates, ROS production is often significantly increased [42]. When ROS levels exceed the capacity of the cellular antioxidant defenses, DNA damage occurs [27]. DNA damage, particularly DSBs, triggers the DNA damage repair mechanisms, primarily mediated by the ATM/ATR kinase pathways, leading to phosphorylation of γH2AX and recruitment of DNA repair factors [43]. ROS-induced DNA damage not only disrupts cellular genomic stability but also accelerates tumor cell death by activating apoptotic pathways [44]. This process is of significant importance in cancer therapy, particularly in enhancing treatment efficacy by increasing ROS production. In our study, we found that vinburnine induces ROS production, which activates the ATM/ATR signaling pathway and leads to phosphorylation of γH2AX, marking the occurrence of DNA double-strand breaks. Further investigations revealed that this DNA damage triggers the activation of apoptosis. Our findings suggest that vinburnine, through its ROS-induced DNA damage pathway, may offer new insights and potential clinical applications for melanoma treatment.

Previous studies have demonstrated that the P38/MAPK signaling pathway is essential for cellular responses to environmental stress, cytokine signals, and DNA damage. The activation of P38 is closely linked to several biological processes, including cell fate determination, DNA repair, cell cycle arrest, and apoptosis. In particular, in tumor cells, this pathway enhances stress responses by modulating the expression of specific transcription factors [45, 46]. ATF3, a key transcription factor activated by stress, is considered a critical downstream target of the P38/MAPK pathway [29]. For instance, Wu et al. reported that IL-33/ST2 suppresses the ATF3-mediated transcriptional downregulation of SLC7A11 through the P38/JNK signaling pathway, thereby modulating ferroptosis [47]. Similarly, Shi et al. demonstrated that arsenic exposure rapidly induces ATF3 expression via the activation of the P38 and JNK pathways [48]. These findings highlight the critical function of the P38/MAPK/ATF3 axis in stress response and transcriptional regulation. In our study, transcriptomic analysis revealed significant enrichment of MAPK pathway-related genes following vinburnine treatment. Subsequent validation experiments showed that P38 was significantly activated, whereas JNK and ERK exhibited no notable changes, aligning with the reported sensitivity of P38 to stress signals. Furthermore, Vinburnine treatment markedly upregulated ATF3 expression, consistent with the transcriptomic findings, and provided further support for P38 as an upstream regulator of ATF3. These results not only corroborate prior studies but also offer novel evidence suggesting that vinburnine exerts its antitumor effects through the activation of the P38/MAPK/ATF3 pathway.

ATF3 is a key regulator of cell fate determination. Previous studies have demonstrated that ATF3 regulates apoptosis and the expression of various immune-related genes under both physiological and pathological conditions [49]. For instance, ATF3 directly interacts with the promoters of DR5 and Bcl-xL to influence apoptosis, while its upregulation in response to arsenic exposure suppresses inflammation by reducing the production of cytokines such as IL-6, IL-8, and TNF-α [48, 50]. These studies highlight the pivotal role of ATF3 in regulating apoptosis and immune responses. In the present study, transcriptomic analysis revealed a notable increase in IL-24 expression following vinburnine treatment. Based on the established transcriptional regulatory functions of ATF3, we hypothesized that IL-24 might be a direct target gene of ATF3. Prior to this investigation, there was no direct evidence linking ATF3 to the regulation of IL-24. To address this gap, we conducted dual-luciferase reporter assays and chromatin immunoprecipitation experiments. Our findings provided the first direct evidence that ATF3 binds to the IL-24 promoter and that vinburnine treatment markedly enhances this interaction. These findings suggest that ATF3 directly regulates IL-24 transcription and contributes to the antitumor effects of vinburnine.

IL-24, a cytokine from the IL-10 family with tumor-suppressive properties, was initially identified in studies on melanoma differentiation-associated gene-7 (MDA-7) [51]. Studies have shown that the expression of IL-24 is markedly diminished in advanced melanoma, with its levels being almost undetectable in metastatic cases, which aligns with its role as a tumor suppressor [52]. IL-24 exerts multifaceted antitumor effects by inducing endoplasmic reticulum stress and ROS generation, thereby promoting tumor cell apoptosis [53, 54], and inhibiting angiogenesis [55]. Notably, IL-24 exhibits selective toxicity toward tumor cells with minimal effects on normal tissues, underscoring its potential for clinical application [56, 57]. Despite the promising efficacy of recombinant IL-24 (rIL-24) in antitumor therapies, its clinical application is hindered by the low efficiency of tumor-targeted delivery. Viral vectors and lipid-based nanoparticle systems have been explored to improve delivery; however, even with intratumoral injection, it remains challenging to ensure effective adenoviral infection of all tumor cells within a lesion [58]. While IL-24-based therapies have progressed to phase I/II clinical trials for various advanced cancers (ClinicalTrials.gov Identifier: NCT00116363), significant advancements in clinical outcomes have yet to be achieved. Compared to traditional IL-24 therapies (such as viral vectors or recombinant protein delivery), our study proposes an innovative strategy that activates endogenous IL-24 expression through a natural product, demonstrating distinct advantages. First, this approach circumvents the technical challenges associated with exogenous protein or gene delivery, reducing the risk of immune responses or off-target toxicity induced by the delivery vectors. Second, by enhancing the expression of endogenous IL-24 locally within tumors, this strategy may effectively reshape the immune microenvironment, potentially synergizing with immune checkpoint inhibitors to further enhance antitumor efficacy.

Boosting T-cell activity is a critical approach for reshaping the TME and enhancing anti-tumor immunity. T-cell activation involves three main signals: First, the T-cell receptor (TCR) recognizes tumor antigens presented by major histocompatibility complex (MHC) molecules, initiating antigen-specific activation. Second, co-stimulatory signals, like the interaction between CD28 and CD80/CD86, are essential for amplifying T-cell proliferation and function. Without this second signal, T-cells may become anergic or ineffective [59]. Third, cytokines play a vital role in the activation, survival, and differentiation of T-cells [60]. For example, IL-2, the first FDA-approved cytokine for advanced melanoma and renal cell carcinoma, supports T-cell survival and expansion [61]. Similarly, IL-12 enhances CD8^+^ T-cell function via its receptor IL-12R, and IL-18 activates T-cells and innate lymphoid cells through the MyD88 pathway [62, 63]. IL-24, a tumor suppressor cytokine, plays a dual role by directly killing cancer cells and modulating the immune microenvironment to amplify antitumor responses [64, 65]. IL-24 binds to receptors of the IL-20 family (IL-20R1/IL-20R2 or IL-22R1/IL-20R2), triggering the JAK/STAT signaling pathway and enhancing CD8⁺ T-cell cytotoxicity [66]. In this study, we demonstrated that vinburnine promotes IL-24 secretion from melanoma cells, leading to enhanced T-cell activity through IL-20R2. We focused on IL-20R2 as it represents the common and indispensable subunit in both IL-24 receptor complexes, thus serving as a critical mediator of IL-24–driven signaling. Consequently, targeting IL-20R2 is expected to broadly influence IL-24–mediated antitumor immunity, independent of the pairing subunit. Nevertheless, we acknowledge that the potential contributions of IL-20R1 and IL-22R1 cannot be excluded. Future studies will be needed to delineate the precise roles of these additional receptor subunits in vinburnine-induced IL-24 signaling, which may provide deeper mechanistic insight and inform the development of more refined therapeutic strategies. Importantly, our results further showed that IL-24 synergizes with anti-PD-1 therapy to enhance antitumor immunity, underscoring its translational potential. Collectively, these findings highlight vinburnine as a promising candidate for melanoma treatment and suggest that targeting the IL-24/IL-20R2 axis may offer broader therapeutic benefits across multiple cancer types, particularly in combination with immune checkpoint inhibitors.

## Conclusions

In summary, our findings show that vinburnine, a compound with multifaceted antitumor activity, induces ROS generation in melanoma cells, leading to apoptosis. Moreover, it activates the P38/MAPK/ATF3 signaling pathway to stimulate IL-24 secretion by melanoma cells, which contributes to reshaping the immunosuppressive TME and enhancing CD8^+^ T cell-mediated immune responses (Fig. 7). Notably, the combination of vinburnine with anti-PD-1 therapy demonstrates a synergistic effect, highlighting its potential as a novel therapeutic approach for melanoma treatment.

## Supplementary Information

Below is the link to the electronic supplementary material.

Supplementary Material 1

## Data Availability

The data that support the findings of this study are available from the corresponding author upon reasonable request.

## Abbreviations

TME: Tumor microenvironment
DDR: DNA damage response
DEGs: Differentially expressed genes
DSBs: Double-strand breaks
ROS: Reactive oxygen species
