# Supplementary material for: Vinburnine potentiates anti-PD1 immunotherapy in melanoma through IL-24 secretion via P38/MAPK/ATF3 signaling
Source: J Exp Clin Cancer Res. 2025 Aug 27;44:255. doi: 10.1186/s13046-025-03521-5 (PMC12382293; doi:10.1186/s13046-025-03521-5)
Supplement: Supplementary file 1 — Supplementary Material 1 [file 13046_2025_3521_MOESM1_ESM.docx]

Supporting Information

Vinburnine Potentiates Anti-PD1 Immunotherapy in Melanoma through IL-24 Secretion via P38/MAPK/ATF3 Signaling

Susi Zhu^a,b,c,d,e,†^ Xu Zhang ^a,b,c,d,e,†^ Waner Liu ^a,b,c,d,e,^ Zhe Zhou ^a,b,c,d,e,^ Siyu Xiong ^a,b,c,d,e,^ Xiang Chen ^a,b,c,d,e,*,^ Cong Peng ^a,b,c,d,e,*^

^a^ Department of Dermatology, Xiangya Hospital, Central South University, Changsha, Hunan, China

^b^ National Engineering Research Center of Personalized Diagnostic and Therapeutic Technology, Xiangya Hospital, Central South University, Changsha, Hunan, China

^c^ Furong Laboratory, Central South University, Changsha, Hunan, China

^d^ Hunan Key Laboratory of Skin Cancer and Psoriasis, Human Engineering Research Center of Skin Health and Disease, Xiangya Hospital, Central South University, Changsha, Hunan, China

^e^ National Clinical Research Center for Geriatric Disorders, Xiangya Hospital, Central South University, Changsha, Hunan, China

^*^Corresponding authors. Tel: +86-172-6749-3866

Email addresses: pengcongxy@csu.edu.cn (Cong Peng), chenxiangck@126.com (Xiang Chen)

^†^ These authors made equal contributions to this work.


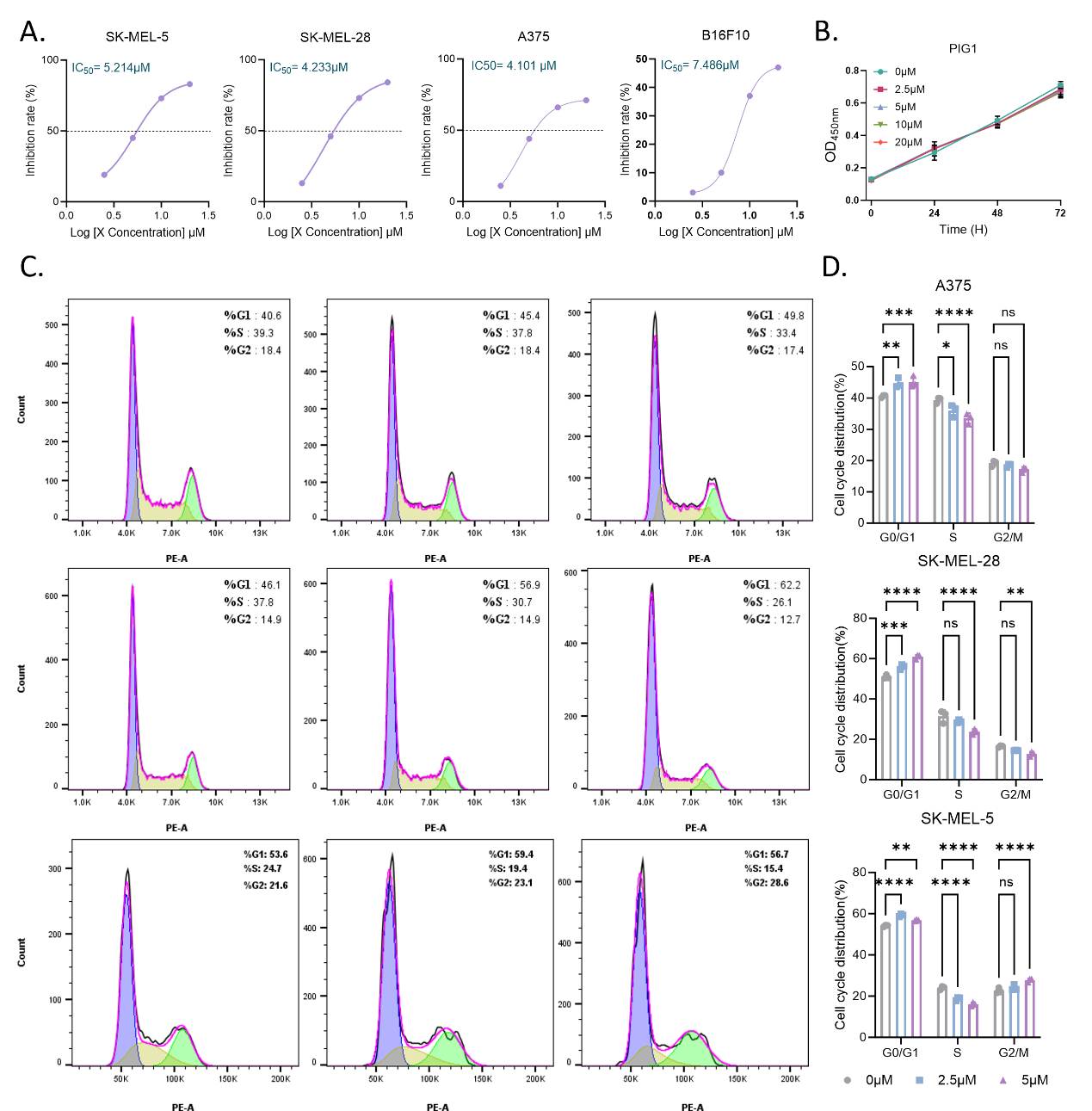


**Figure S1.** Vin inhibits melanoma proliferation and cell cycle progression. A) Determination of the IC50 value of Vin in SK-MEL-5, SK-MEL-28, and A375 cells after 48 hours. B) Proliferation of PIG1 cells was assessed by CCK-8 assay at 24, 48, and 72 hours following treatment with Vin (2.5, 5, 10, 20 μM). C) Flow cytometry analysis of cell cycle distribution in A375, SK-MEL-28, and SK-MEL-5 cells treated with 2.5 or 5 μM Vin for 24 hours. D) Quantification of the G0/G1, S, and G2/M phases in A375 and SK-MEL-28 cells. n=3. Data were presented as means ± SD, ns., not significant, *∗*P < 0.05, *∗∗*P < 0.01, *∗∗∗*P < 0.001 according to two-way ANOVA.


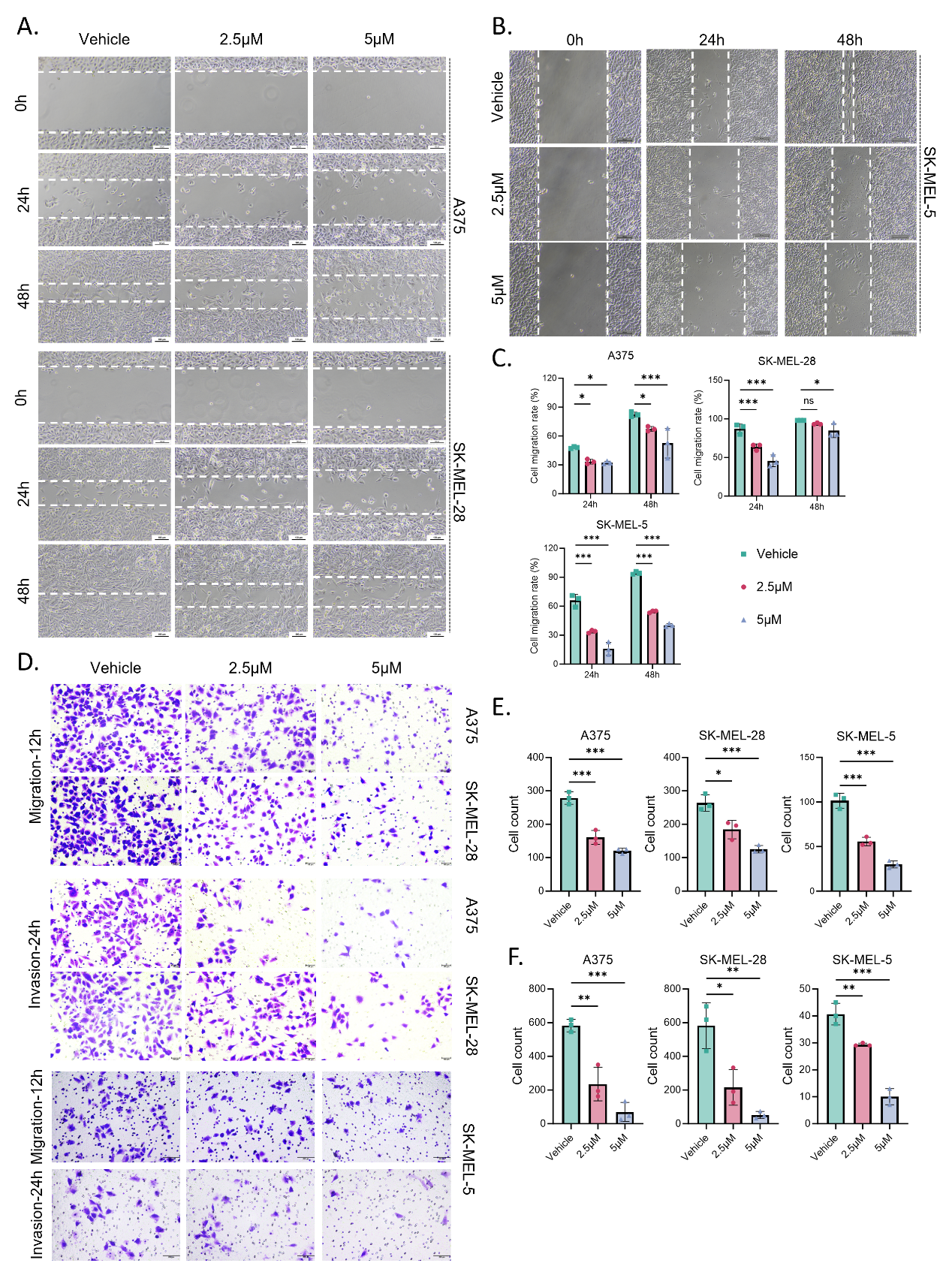


**Figure S2** Vin inhibits melanoma migration and invasion. A-C) Wound healing assay evaluating the migration of A375, SK-MEL-28, and SK-MEL-5 cells treated with 2.5 and 5 μM of Vin for 24 and 48 hours. n=3. Data were presented as means ± SD, ns., not significant, *∗*P < 0.05, *∗∗*P < 0.01, *∗∗∗*P < 0.001 according to two-way ANOVA. Scale bars, 100μm. D,E) Transwell migration and invasion assays assessing the effects of 2.5 and 5 μM of Vin on A375 and SK-MEL-28 cells migration and invasion. n=3. Data were presented as means ± SD, *∗*P < 0.05, *∗∗*P < 0.01, *∗∗∗*p < 0.001 according to two-way ANOVA. Scale bars, 100μm.


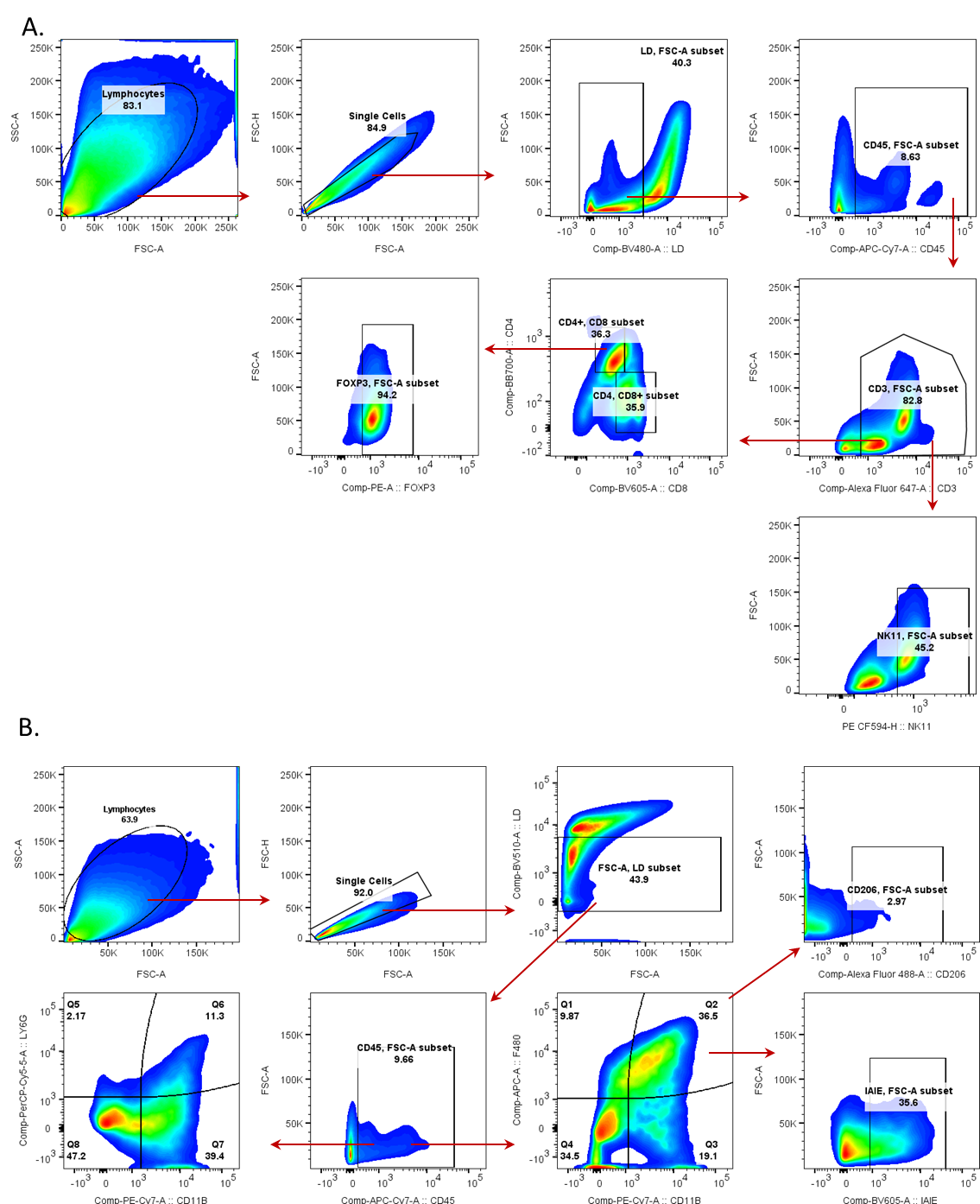


**Figure S3.** Flowchart of the gating strategy used for flow cytometry analysis. A) T cell and NK cell panel. B) Myeloid cell panel.


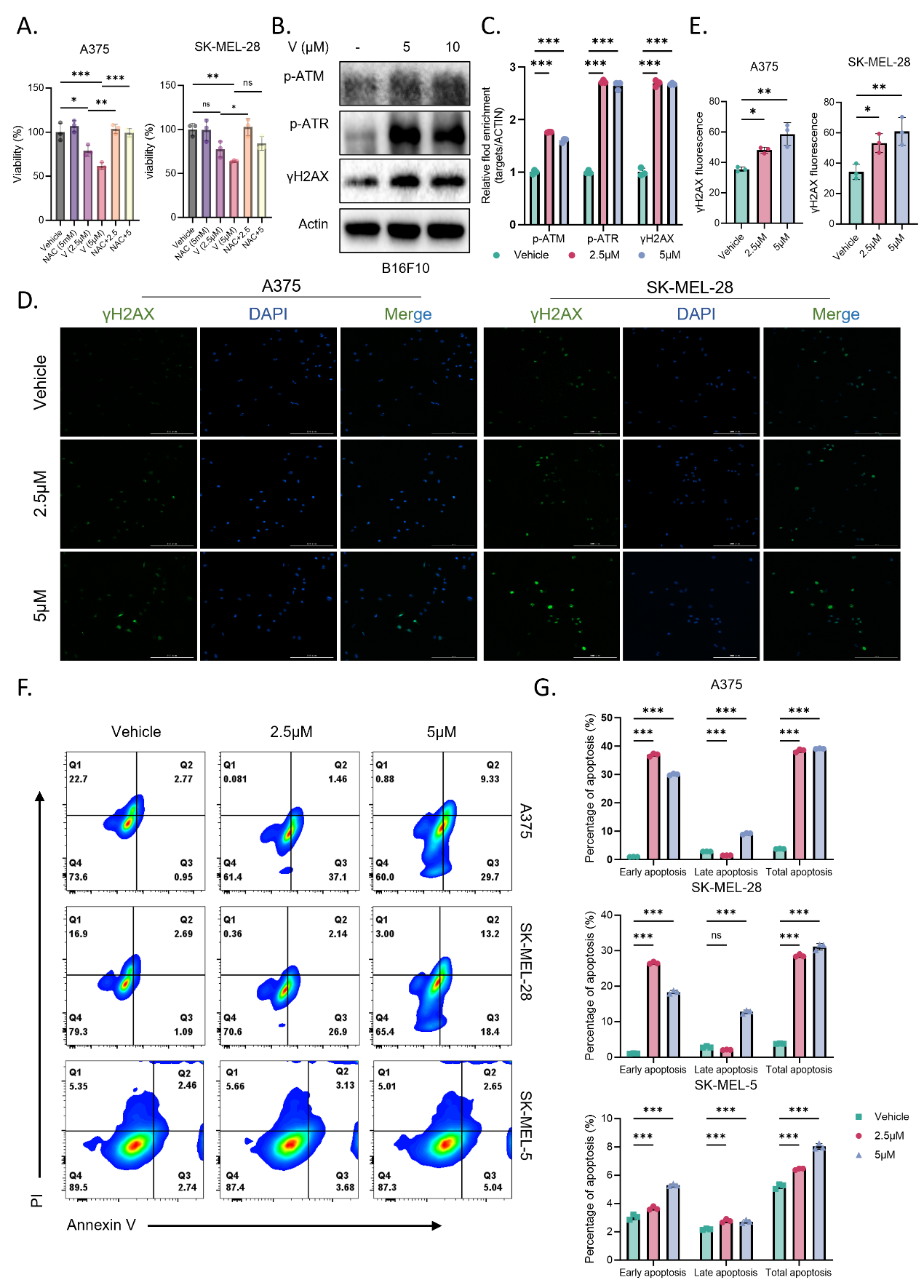


**Figure S4.** A) CCK-8 assay to measure cell viability of A375 or SK-MEL-28 cells treated with 5 mM ROS inhibitor NAC combined with 2.5 μM or 5 μM Vin for 24 hours. n=3. Data were presented as means ± SD, ns., not significant, *∗*P < 0.05, *∗∗*P < 0.01, *∗∗∗*P < 0.001 according to two-way ANOVA. B) Western blot analysis of changes in DNA damage markers p-ATM, p-ATR, and γH2AX in B16F10 cells treated with 5μM or 10 μM Vin for 48 hours. C) Quantification of western blot band intensities shown in (B), n=3. Data were presented as means ± SD, *∗∗∗*P < 0.001 according to two-way ANOVA. D) Immunofluorescence analysis of γH2AX expression in A375 and SK-MEL-28 cells treated with 2.5 μM or 5 μM Vin for 48 hours. E) Quantitative immunofluorescence analysis of γH2AX expression shown in (D), n=3. Data were presented as means ± SD, *∗*P < 0.05, *∗∗*P < 0.01, according to two-way ANOVA. F, G) Flow cytometric analysis of apoptosis levels in A375, SK-MEL-28, and SK-MEL-5 cells treated with 2.5 and 5 μM of Vin. n=3. Data were presented as means ± SD, ns., not significant, ∗∗∗P < 0.001 according to two-way ANOVA.


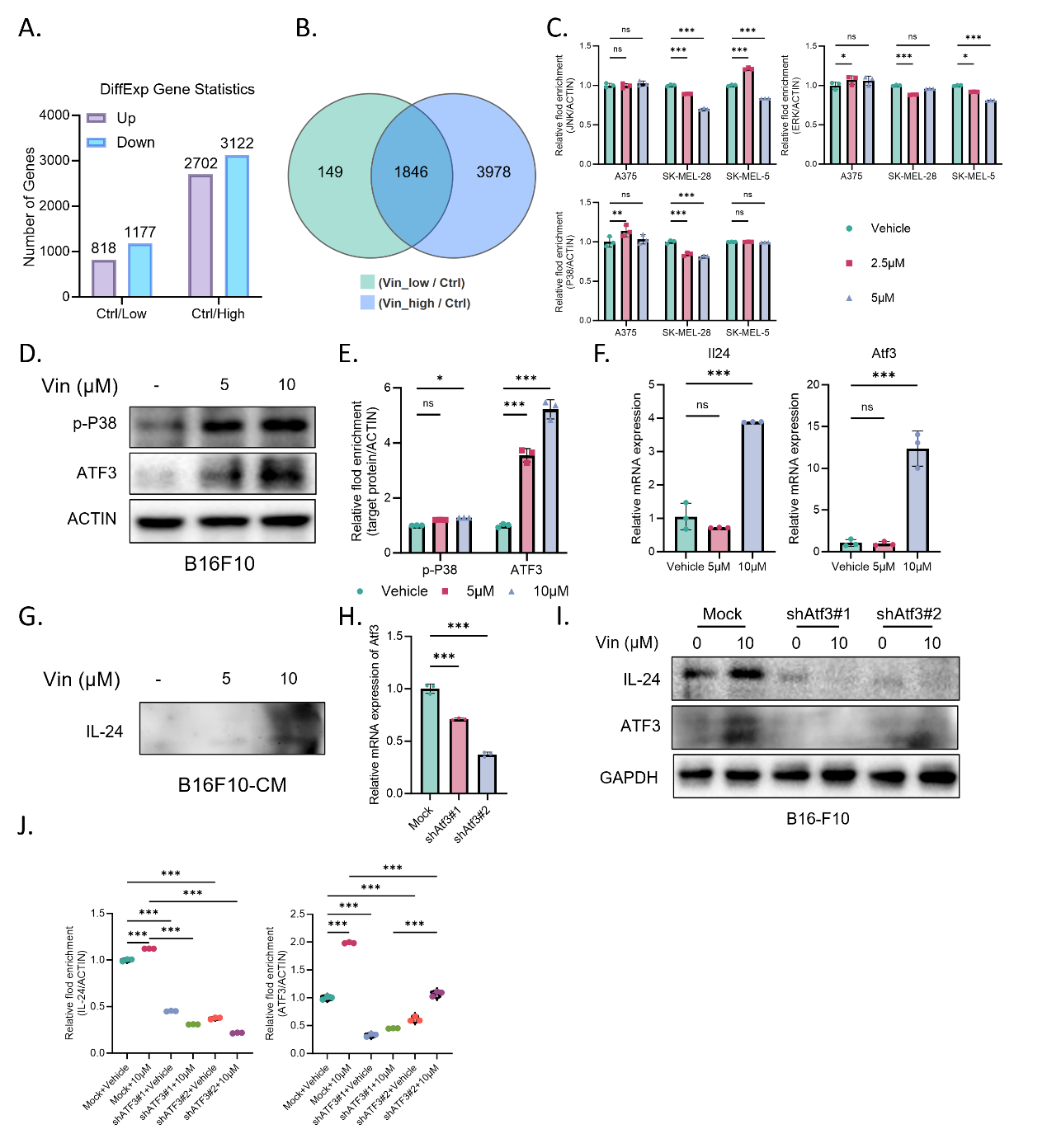


**Figure S5.** Vin upregulates IL-24 in melanoma by activating the p-P38/MAPK/ATF3 signaling axis. A) Bar chart showing DEGs from RNA sequencing. B) Venn diagram showing the common differentially expressed genes between the high-dose and low-dose groups from RNA sequencing. C) Quantification of western blot band intensities shown in (Figure 3B), n=3. Data were presented as means ± SD, ns., not significant, *∗*P < 0.05, *∗∗*P < 0.01, *∗∗∗*P < 0.001 according to two-way ANOVA. D) Western blot analysis of p-P38 and ATF3 protein levels in B16F10 cells treated with 5 μM or 10 μM Vin for 48 hours. E) Quantification of p-P38 and ATF3 protein levels relative to ACTIN from panel C), n=3. Data were presented as means ± SD, ns., not significant, *∗*P < 0.05, *∗∗∗*P < 0.001 according to two-way ANOVA. F) RT-PCR analysis of IL-24 and Atf3 mRNA levels in B16F10 cells treated with 5 μM or 10 μM Vin for 48 hours, n=3. Data were presented as means ± SD, ns., not significant, *∗*P < 0.05, *∗∗∗*P < 0.001 according to two-way ANOVA. G) Western blot measurement of IL-24 levels in the conditioned medium from B16F10 cells treated with 5 or 10 μM of Vin for 48 hours. H) RT-PCR analysis of Atf3 mRNA expression in ATF3 knockdown B16F10 cells, n=3. Data were presented as means ± SD, *∗∗∗*P < 0.001 according to two-way ANOVA. I) Western blot analysis of ATF3 and IL-24 expression in B16F10 cells with control or ATF3 knockdown, treated with 10 μM Vin for 48 hours. J) Quantification of IL-24 and ATF3 protein levels relative to ACTIN from panel I). Bars, mean ± SD; *∗∗∗P* < 0.001 according to two-way ANOVA.


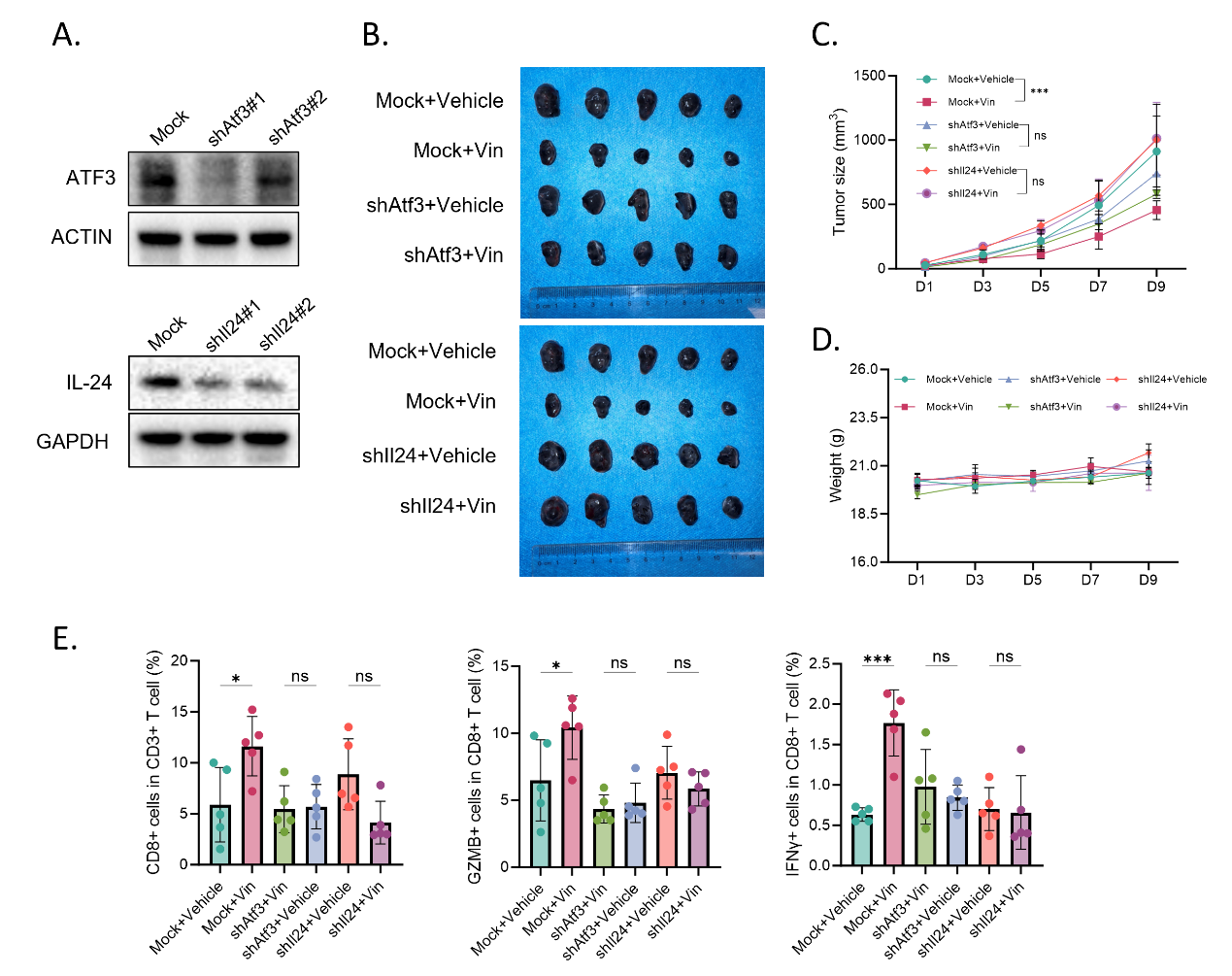


**Figure S6.** Validation of shATF3 and shIL24 knockdown efficiency and antitumor immune responses in vivo. A) Western blot analysis showing knockdown efficiency of ATF3 and IL-24 in B16F10 cells. B) Representative images of dissected B16F10 tumors from C57BL/6 mice after indicated treatments. C) Tumor growth curves of B16F10 tumors in C57BL/6 immune-competent mice treated with the indicated agents (n = 5). Data are presented as means ± SD; ns, not significant; *∗∗∗*p < 0.001 by one-way ANOVA. D) Body weight of mice monitored throughout the treatment period, showing no significant differences. E) Flow cytometry analysis of tumor-infiltrating CD8⁺ T cells, GZMB⁺CD8⁺ T cells, and IFNγ⁺CD8⁺ T cells (n = 5). Data are presented as means ± SD; ns, not significant; *∗*p < 0.05, *∗∗∗*p < 0.001 by one-way ANOVA.


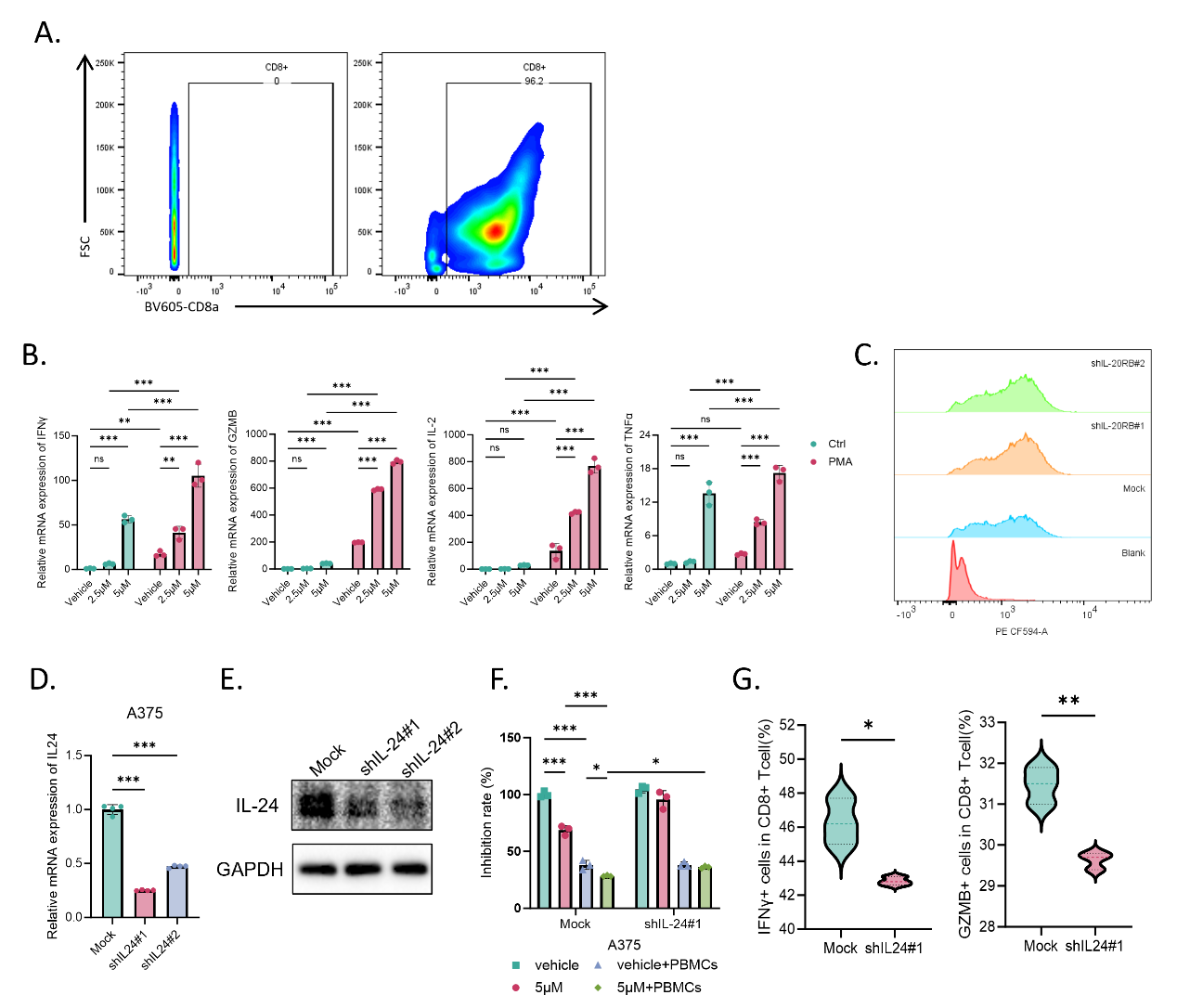


**Figure S7.** Vin enhances CD8+ T cell cytotoxicity by promoting IL-24 secretion in melanoma. A) Flow cytometry analysis of the sorting efficiency of CD8^+^ T cells isolated by magnetic bead separation. B) A375 cells were treated with 2.5 μM or 5 μM Vin for 48 hours, followed by a 48-hour incubation in fresh complete medium. The collected supernatant was then used to treat activated or non-activated Jurkat cells. RT-PCR was performed to measure the mRNA levels of IFNγ, GZMB, IL-2, and TNFα. n=3, Bars, mean ± SD; ns: not significant; *∗∗P* < 0.01; *∗∗∗P* < 0.001. C) Flow cytometry sorting of Jurkat cells infected with lentivirus carrying cherry-tagged IL-20R2 knockdown. D) RT-PCR analysis of IL-24 knockdown efficiency in A375 cells. n=4, Bars, mean ± SD; *∗∗∗P* < 0.001; *∗∗∗∗P* < 0.0001. E) Western blot analysis showing knockdown efficiency of IL-24 in A375 cells. F) Luciferase reporter assay to measure the inhibition rate of control or IL-24 knockdown A375 cells treated with 5 μM Vin and PBMCs. n=3, Bars, mean ± SD; *∗P* < 0.05; *∗∗∗P* < 0.001. G) Flow cytometry analysis of the proportion of IFNγ^+^CD8^+^ T cells or GZMB^+^CD8^+^ T cells in PBMCs co-cultured with control or IL-24 knockdown A375 cells. Bars, mean ± SD; *∗P* < 0.05; *∗∗P* < 0.01, according to paired t-test.


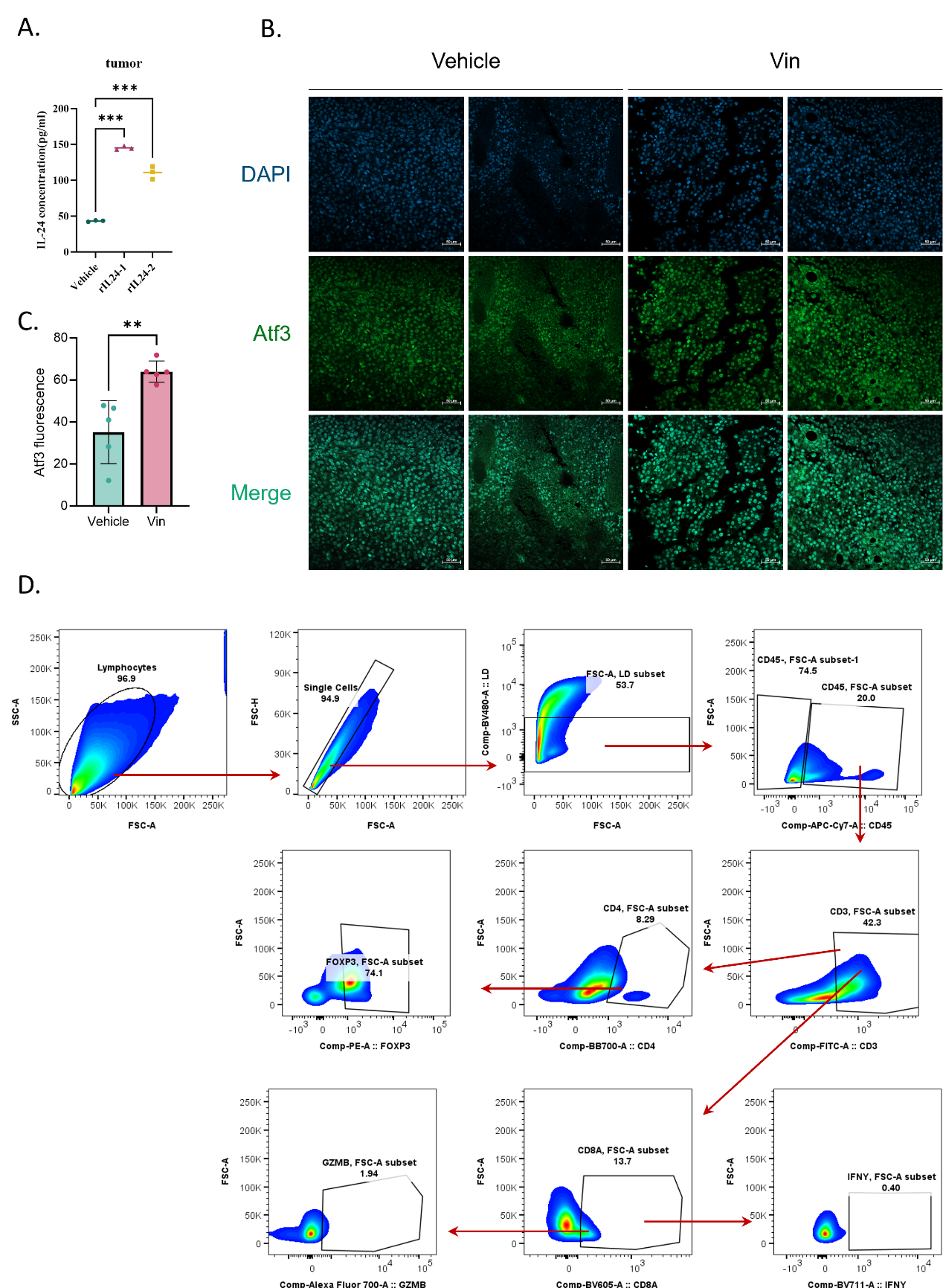


**Figure S8.** The combination of Vin and PD-1 blockage suppresses melanoma growth. A) ELISA analysis of IL-24 levels in tumor tissues 24 hours after intratumoral injection of rIL-24 in B16F10-bearing mice. n=5. Data were presented as means ± SD, *****p < 0.001 by one-way ANOVA. B,C) Immunofluorescence analysis of ATF3 expression in tumor tissues from vehicle- and Vin-treated (5 mg/kg) mice. n=5. Data were presented as means ± SD, *∗∗*p < 0.01, according to paired t-test. D) Flowchart of the gating strategy for IFNγ and GZMB detection by flow cytometry.


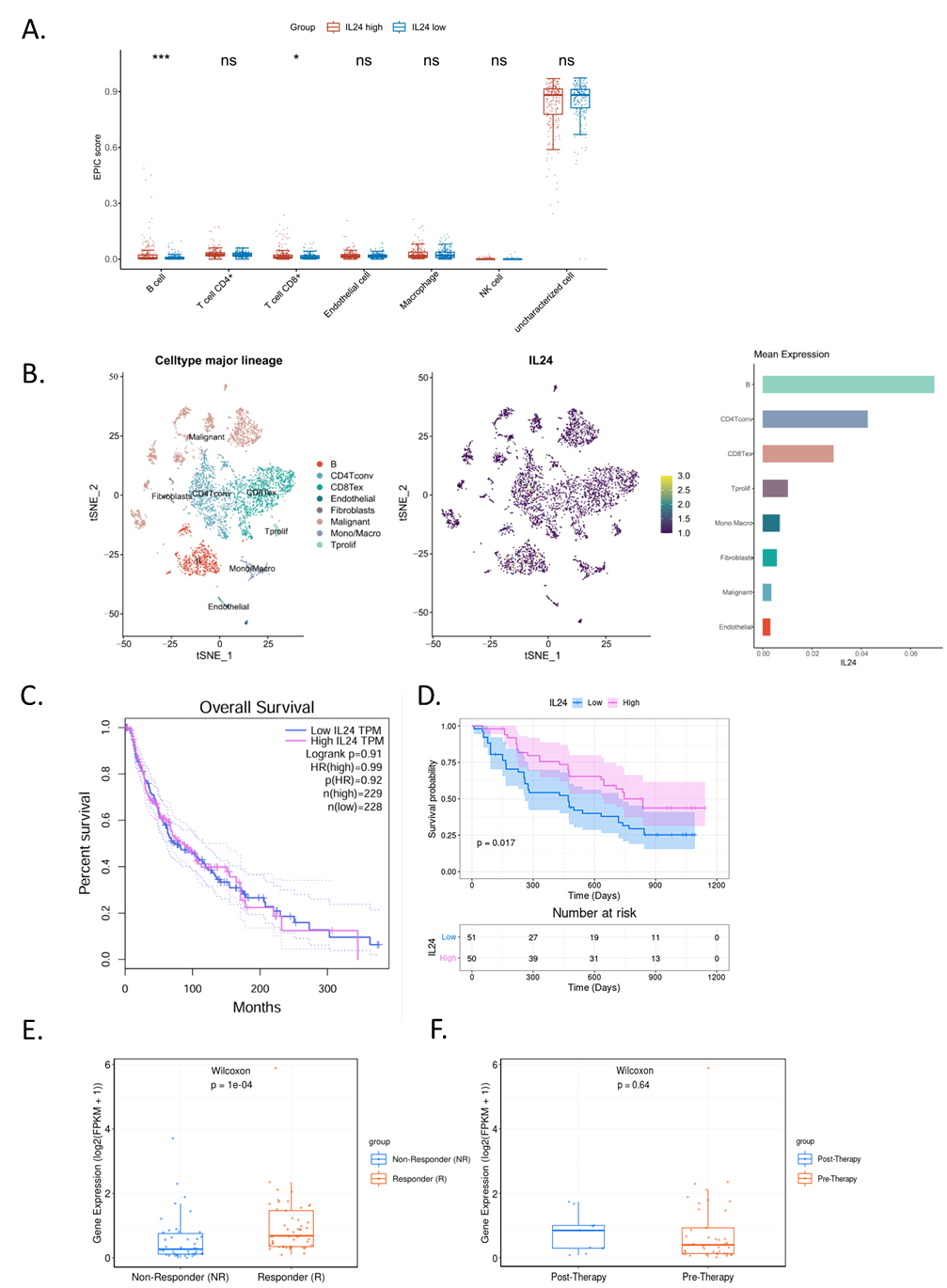


**Figure S9.** Correlation of IL-24 expression with immune infiltration, anti-PD-1 response, and prognosis in melanoma. A) Distribution of immune scores in melanoma with high (n=235) vs. low (n=236) IL-24 expression based on TCGA database. The x-axis represents the types of immune cell infiltration, and the y-axis shows the distribution of immune infiltration scores across different tissues. Statistical significance between the two groups was assessed using the Wilcoxon rank-sum test. B) The t-SNE plot of single-cell clustering from the SKCM_GSE72056 dataset, with different colors representing different cell types (left). The t-SNE plot showing the distribution of IL-24 expression across different cell types, with color intensity representing expression levels. Darker colors indicate lower expression, while brighter colors represent higher expression of IL-24 in the cells (middle). Bar chart showing the expression levels of IL-24 across different cell types (right). C) Overall Survival Analysis of IL-24 in SKCM using GEPIA Database. D) Analysis of the correlation between IL-24 expression and overall survival in melanoma patients treated with anti-PD-1 therapy, based on the GSE91061 dataset from the TIGER database. E, F) Analysis of IL-24 expression in melanoma patients who responded or did not respond to anti-PD-1 therapy E), as well as the expression of IL-24 before and after anti-PD-1 treatment F), based on the PRJEB23709 dataset from the TIGER database.

**Supplementary** **Table 1a (Human)**

| Gene | Forward | Reverse |
| --- | --- | --- |
| IL-24 | AGCTCAGGATAACATCACGAGTG | CTGTGTGCACTGTCTCTGATGG |
| ATF3 | CCTCTGCGCTGGAATCAGTC | TTCTTTCTCGTCGCCTCTTTTT |
| TNFα | TGGCCCAGACCCTCACACTCAG | ACCCATCGGCTGGCACCACT |
| IL-2 | GCATTTACTGCTGGATTT | ATGTTTCAGTTCTGTGGC |
| IFNγ | GGAGGAACTGGCAAAAGGAT | TTCAAGACTTCAAAGAGTCTGAGG |
| GZMB | CCCTGGGAAAACACTCACACA | GCACAACTCAATGGTACTGTCG |
| IL20R2 | GGCCACTGTGCCATACAAC | TCTTTGGTGATCTCCATCCCA |
| IL-12a | CCTTGCACTTCTGAAGAGATTGA | ACAGGGCCATCATAAAAGAGGT |
| IL-32 | TCAAAGAGGGCTACCTGGAG | TTTCAAGTAGAGGAGTGAGCTCTG |
| EBI3 | TCATTGCCACGTACAGGCTC | GGGTCGGGCTTGATGATGTG |
| TNC | TCCCAGTGTTCGGTGGATCT | TTGATGCGATGTGTGAAGACA |
| ACTIN | CCTGGCACCCAGCACAAT | GGGCCGGACTCGTCATAC |

**Supplementary Table 1b (Mouse)**

| Gene | | Forward | Reverse |
| --- | --- | --- | --- |
| Atf3 | GAGGATTTTGCTAACCTGACACC | | TTGACGGTAACTGACTCCAGC |
| Il24 | GAGCCTGCCCAACTTTTTGTG | | TGTGTTGAAGAAAGGGCCAGT |
| Actin | GGCTGTATTCCCCTCCATCG | | CCAGTTGGTAACAATGCCATGT |

**Supplementary Table 2**

| Primer | Forward | Reverse |
| --- | --- | --- |
| Primer1 | taccctgaggccagccaaggtgta | cagggcagctgaaatcctagacta |
| Primer2 | ggagtattttgtttgcttaagaaa | atgaaagaaaagaggagagtgag |
| Primer3 | aatatgtcaacaaacatatgtgac | tgcgacctaagagtagtttttacg |
| Primer4 | gctggattaaacagagttaagaag | ttgaggaattcttgtcttgcaag |
| Primer5 | gttgagtgattataaccctaggga | gctgggacagtagtccacagcgaa |
| Primer6 | cgctgtattgtccttaaagtgatg | gcatatatatgggggctgatgggg |

**Supplementary Table 3 Fluorescence labeled-antibodies used for flow cytometry analysis.**

| **Gene** | **Clone** | **Catalogue** | **Vendor** |
| --- | --- | --- | --- |
| Alexa Fluor® 488 anti-mouse IFN-γ Antibody | XMG1.2 | 505815 | Bioleged |
| Alexa Fluor® 700 anti-human/mouse Granzyme B Recombinant Antibody | QA16A02 | 372221 | Bioleged |
| PerCP/Cyanine5.5 anti-mouse CD4 Antibody | GK1.5 | 100434 | Bioleged |
| APC anti-mouse CD3 Antibody | 17A2 | 100236 | Bioleged |
| PE/Dazzle™ 594 anti-mouse NK-1.1 Antibody | PK136 | 108748 | Bioleged |
| APC/Cyanine7 anti-mouse CD45 Antibody | 30-F11 | 103116 | Bioleged |
| Brilliant Violet 605™ anti-mouse CD8a Antibody | 53-6.7 | 100744 | Bioleged |
| FOXP3 Monoclonal Antibody (FJK-16s), PE | FJK-16s | 12-5773-82 | ebioscience |
| Alexa Fluor® 488 anti-mouse CD206 (MMR) Antibody | C068C2 | 141709 | Bioleged |
| PerCP/Cyanine5.5 anti-mouse Ly-6G/Ly-6C (Gr-1) Antibody | RB6-8C5 | 108428 | Bioleged |
| APC anti-mouse F4/80 Antibody | BM8 | 123116 | Bioleged |
| Brilliant Violet 605™ anti-mouse I-A/I-E Antibody | M5/114.15.2 | 107639 | Bioleged |
| PE/Cyanine7 anti-mouse/human CD11b Antibody | M1/70 | 101216 | Bioleged |
| Alexa Fluor® 700 anti-human CD3 Antibody | OKT3 | 317340 | Bioleged |
| Brilliant Violet 605™ anti-human CD8 Antibody | SK1 | 344741 | Bioleged |
| PE anti-human IFN-γ Antibody | 4S.B3 | 502509 | Bioleged |
| FITC anti-human/mouse Granzyme B Recombinant | QA16A02 | 372206 | Bioleged |
